# MCC-SP: a powerful integration method for identification of causal pathways from genetic variants to complex disease

**DOI:** 10.1186/s12863-020-00899-3

**Published:** 2020-08-26

**Authors:** Yuchen Zhu, Jiadong Ji, Weiqiang Lin, Mingzhuo Li, Lu Liu, Huanhuan Zhu, Fuzhong Xue, Xiujun Li, Xiang Zhou, Zhongshang Yuan

**Affiliations:** 1 Department of Biostatistics, School of Public Health, Cheeloo College of Medicine, Shandong University, Jinan, 250012 Shandong China; 2 Department of Data Science, School of Statistics, Shandong University of Finance and Economics, Jinan, 250014 China; 3 Department of Biostatistics, University of Michigan, Ann Arbor, MI 48109 USA; 4 Center for Statistical Genetics, University of Michigan, Ann Arbor, MI 48109 USA

**Keywords:** Maximum correlation coefficient, K shortest paths algorithms, Integration method, Pathway, Alzheimer’s disease

## Abstract

**Background:**

Genome-wide association studies (GWAS) have successfully identified genetic susceptible variants for complex diseases. However, the underlying mechanism of such association remains largely unknown. Most disease-associated genetic variants have been shown to reside in noncoding regions, leading to the hypothesis that regulation of gene expression may be the primary biological mechanism. Current methods to characterize gene expression mediating the effect of genetic variant on diseases, often analyzed one gene at a time and ignored the network structure. The impact of genetic variant can propagate to other genes along the links in the network, then to the final disease. There could be multiple pathways from the genetic variant to the final disease, with each having the chain structure since the first node is one specific SNP (Single Nucleotide Polymorphism) variant and the end is disease outcome. One key but inadequately addressed question is how to measure the between-node connection strength and rank the effects of such chain-type pathways, which can provide statistical evidence to give the priority of some pathways for potential drug development in a cost-effective manner.

**Results:**

We first introduce the maximal correlation coefficient (MCC) to represent the between-node connection, and then integrate MCC with K shortest paths algorithm to rank and identify the potential pathways from genetic variant to disease. The pathway importance score (PIS) was further provided to quantify the importance of each pathway. We termed this method as “MCC-SP”. Various simulations are conducted to illustrate MCC is a better measurement of the between-node connection strength than other quantities including Pearson correlation, Spearman correlation, distance correlation, mutual information, and maximal information coefficient. Finally, we applied MCC-SP to analyze one real dataset from the Religious Orders Study and the Memory and Aging Project, and successfully detected 2 typical pathways from APOE genotype to Alzheimer’s disease (AD) through gene expression enriched in Alzheimer’s disease pathway.

**Conclusions:**

MCC-SP has powerful and robust performance in identifying the pathway(s) from the genetic variant to the disease. The source code of MCC-SP is freely available at GitHub (https://github.com/zhuyuchen95/ADnet).

## Background

Over the last decade, genome-wide association studies (GWAS) have achieved remarkable successes in identifying genetic susceptible variants (e.g. SNPs, Single Nucleotide Polymorphism) for a variety of complex traits or diseases [1]. However, the underlying biological pathway mechanism of such association remains largely unknown. Indeed, the genetic variant can act on other molecular traits (e.g. gene expression) and they together weave into one biological network or pathway that contributes to a disease. For instance, the *APOE* gene has been well identified to be associated with Alzheimer’s disease from large scale GWAS [2–5], one possible explanation is that the SNP variant in *APOE* can first regulate the *APOE* gene expression [6–10], then act on the production of amyloid plaques and neurofibrillary tangles, and finally lead to AD [11–13].

Most disease-associated genetic variants from GWAS have been shown to lie in noncoding regions across the genome [1, 14, 15], which provides the clues that regulation of gene expression levels may be the primary biological mechanism through which genetic variants affect complex disease. Certainly, the top GWAS SNP can be also significantly detected due to the linkage disequilibrium with the true causal one, which could be an exonic variant [16]. In addition, several expression quantitative trait loci (eQTLs) studies also illustrate that the expression regulatory information may play a pivotal role in bridging the gap between genetic variants and traits [17–19]. Up until now, there are a few methods to characterize the gene expression that mediates the effect of the genetic variant on complex disease. Huang et al. proposed a model to exploit gene expression to more powerfully test the association between SNPs and diseases by jointly modeling SNPs, gene expressions and diseases [20, 21]. Recent transcriptome-wide association studies (TWAS) have been widely used to integrate the expression regulatory information from eQTL studies with GWAS data to identify gene expression that links the cis-SNPs (SNPs that are within a predefined gene or other well-defined genetic region) and the complex disease [22–24]. Nevertheless, these studies commonly analyzed one gene at a time, while the genetic variant can affect the complex disease through multiple genes and multiple pathways. Park et al. developed the causal multivariate mediation within extended linkage disequilibrium (CaMMEL) method in Bayesian inference framework to select target genes mediating the effect of genetic variants on the complex disease [25]. Wei and Li proposed the nonparametric pathways-based regression (NPR) that can consider multiple pathways simultaneously and allow complex interactions among genes within the pathways [26]. Yao et al. developed a model to quantify the proportion of disease heritability that is specifically mediated in *cis* region by the assayed expression levels of the set of all genes, and of genes in specific functional categories [27]. Indeed, it has been well documented in GWAS that the multiple gene or pathway-based approach can improve power [28]. Although these methods include multiple gene expression in the model, they ignore the complex network structure relationship among genes, which, from the network medicine perspective, is hard to investigate the precise network or molecular pathways involved in complex disease. In fact, one single gene expression can express some mediated effects from the SNP variant to the disease when studying it alone, while this effect could change substantially when studying it within one network or pathway, and vice versa [29, 30]. The focus has been shifted to the identification of pathways.

Often, there could be multiple pathways from the SNP variant to the final disease, with each having the chain structure since the first node is specific SNP variant and the end outcome is the disease. One key but inadequately addressed question is, given such chain pathway, how to measure the connection strength between two nodes and detect whether such pathway is the potential causal one. It is highly desirable to develop statistical methods for ranking the effect of these pathways, providing evidence to give the priority of some pathways for potential drug development and offer drug targets in a cost-effective and timely manner. In the context of systems epidemiology, Ji et al. developed a statistic to test the pathway effect that contributed to a disease with a case-control design [31]. Yuan et al. proposed a novel chi-square statistic to identify whether one chain-type pathway is associated with the final disease [30]. However, their methods simply use the linear regression to represent the between-node correlation, which is insufficient to capture the complex dependency between the nodes. Furthermore, their methods did not include the outcome variable (complex trait or disease) into the pathway and cannot essentially investigate the pathway mechanism. For example, if there is one potential pathway SNP → gene expression 1 → gene expression 2 → AD, their methods, under linear between-node correlation, can detect SNP → gene expression 1 → gene expression 2 is significantly associated with AD, but failed to determine whether AD is connected to gene expression 1 or gene expression 2. Given that the goal is to rank and quantify the effect of the pathways from the genetic variant to the complex disease, it is intuitively to put such a question into the framework of graph theory once the suitable quantity is found to measure the connection strength between two nodes in the pathway. At present study, we first introduced the maximal correlation coefficient (MCC) to represent the between-node connection, and then integrate MCC with the commonly used K shortest paths algorithm [32–34] in graph theory to rank and identify the potential pathways from genetic variant to disease. We further defined the pathway importance score (PIS) to quantify the importance of each pathway. We termed this method as “MCC-SP”. Various simulations, with different sample sizes and network structures, are conducted to illustrate MCC is better to measure the between-node correlation than other quantities including Pearson correlation, Spearman correlation, distance correlation, mutual information, and maximal information coefficient. MCC-SP, as an integration method, has always better and robust performance in identifying the causal pathway from genetic variant to the disease. From the Religious Orders Study and the Memory and Aging Project (ROSMAP), we further applied MCC-SP to identify the potential causal pathway from *APOE* genotype to AD through gene expression enriched in Alzheimer’s disease pathway.

## Results

### Simulation

**Fig. 1:**
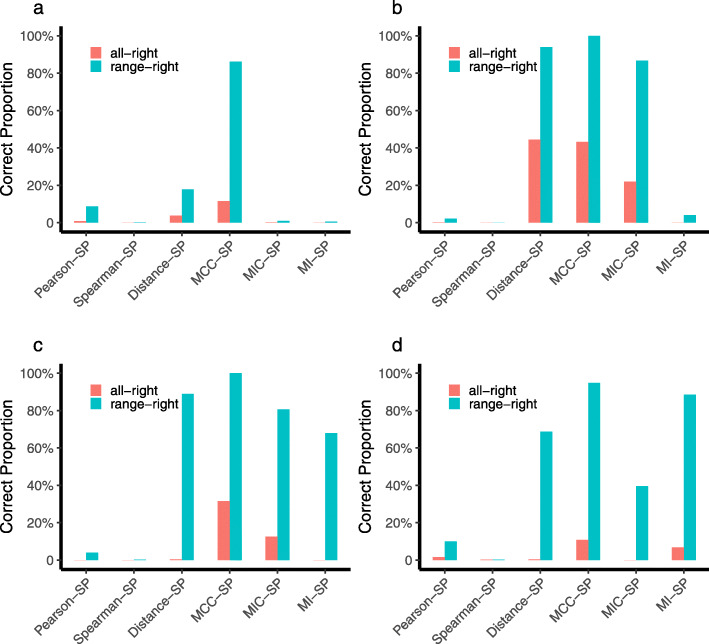
The proportion that correctly pinpoint the top 4 pathways among 500 simulations under two criteria when the sample size is 500 and the proportion of nonlinear components is 40%.The nonlinear pattern is (**a**) $\boldsymbol{\varphi} \left({\boldsymbol{x}}_{\boldsymbol{i}}\right)=\sqrt{\boldsymbol{C}-{\boldsymbol{x}}_{\boldsymbol{i}}^{\mathbf{2}}}+\boldsymbol{\varepsilon}$, (**b**) ***φ***(***x***_***i***_) ***=***  **cos** (***x***_***i***_) ***+ ε***, (**c**) mixed nonlinear pattern(6 edges having cosine and 3 edges having quadratic relationship) and (**d**) $\boldsymbol{\varphi} \left({\boldsymbol{x}}_{\boldsymbol{i}}\right)={\boldsymbol{x}}_{\boldsymbol{i}}^{\mathbf{2}}+\boldsymbol{\varepsilon}$ respectively

**Fig. 2:**
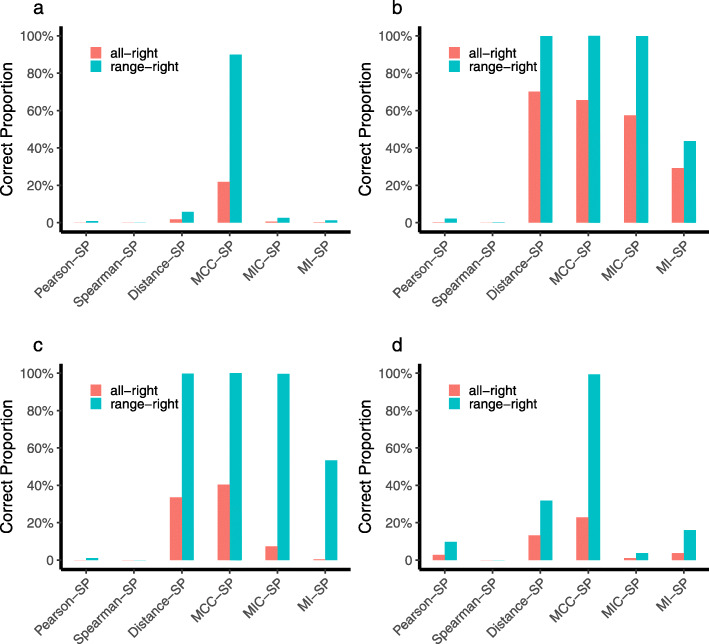
The proportion that correctly pinpoint the top 4 pathways among 500 simulations under two criteria when the sample size is 500 and the proportion of nonlinear components is 50%.The nonlinear pattern is (**a**) $\boldsymbol{\varphi} \left({\boldsymbol{x}}_{\boldsymbol{i}}\right)=\sqrt{\boldsymbol{C}-{\boldsymbol{x}}_{\boldsymbol{i}}^{\mathbf{2}}}+\boldsymbol{\varepsilon}$, (**b**) ***φ***(***x***_***i***_) ***=***  **cos** (***x***_***i***_) ***+ ε***, (**c**) mixed nonlinear pattern(8 edges having cosine and 4 edges having quadratic relationship) and (**d**) $\boldsymbol{\varphi} \left({\boldsymbol{x}}_{\boldsymbol{i}}\right)={\boldsymbol{x}}_{\boldsymbol{i}}^{\mathbf{2}}+\boldsymbol{\varepsilon}$ respectively

Table 1 shows the simulation results when all the between-node correlations are linear. When the sample size is relatively large (e.g. 300, 500), all methods except MI-SP and MIC-SP have comparable performance as the Pearson-SP, which is the gold standard in such case under both all-right and range-right criteria. When the sample size reduced to 100, the superiority of Pearson -SP is more obvious, though the power of all methods decreases. Figures 1 and 2 show the results of all six integration-methods under sample size 500 and 4 different nonlinear correlation patterns being arcuate, cosine, quadratic and mixed pattern. Under the arcuate nonlinear pattern, the MCC-SP performs better than any other method under both criteria regardless of the proportion of nonlinear components (Figs. 1a and 2a). Note that under the all-right criteria, the other methods are unable to identify the top 4 pathways at all. Under cosine nonlinear relationship, both MCC-SP and DC-SP have comparably better performance than that of the other methods (Figs. 1b and 2b), even when the proportion of nonlinear component reached 60% (Figure S1). However, MCC-SP has the best performance under the all-right criteria when the nonlinear proportion is 30% (Figure S2). Similar phenomenon can be found under the sine relationship (Figure S3). Under the mixed nonlinear relationship, MCC-SP performs best under both criteria (Fig. 1c) and have comparable better performance with DC-SP than that of other methods (Fig. 2c). Under the quadratic relationship, MCC-SP still have the best performance than any other methods (Fig. 1d and Fig. 2d). The results are consistent under the exponential relationship or the reciprocal relationship (Figure S4), or when comparing MCC-SP with the nonparametric pathways-based regression (NPR) model (Figure S5).

**Table 1:** The number of times that properly pinpoint the top 4 pathways among 500 simulations with linear between-node correlation

Sample	Criteria	Pearson	Spearman	Distance	MCC	MIC	MI
100	All-right	152	129	125	107	44	1
	Range-right	408	402	428	378	300	46
300	All-right	271	252	248	248	88	0
	Range-right	492	490	495	486	468	218
500	All-right	444	429	415	440	152	254
	Range-right	500	500	500	500	500	500

The simulation results under sample size 100 and 300 are in the Figures S6, S7, S8, S9, S10, S11, S12, S13, S14, S15, where similar phenomenon can be found.

### Real data

Overall, totally 6 pathways from *APOE* genotype to AD have been identified to be top 5 from all the 6 integration methods (Table 2). The findings of each method are inconsistent. Pearson-SP, Spearman-SP, DC-SP and MCC-SP ranked the pathway *APOE* genotype →*APOE* gene expression →*GRIN2A* → *CAPN2* → *MAPT*→AD to be first, which illustrates this pathway may play important roles in the AD mechanism. Both DC-SP and MCC-SP ranked *APOE* genotype →*APOE* gene expression →*GRIN2A* → *NOS1* → AD to be second, and this pathway has been ranked to be first by MIC-SP and MI-SP, which indicates that this pathway may also has a high probability to involve the AD mechanism. Both Spearman-SP and MCC-SP ranked *APOE* genotype →*APOE* gene expression →*CACNA1C* → *CAPN2* → *MAPT*→AD as the third. MCC-SP ranked *APOE* genotype →*APOE* gene expression→*CACNA1C* → *NOS1* → AD as the fourth and *APOE* genotype →*APOE* gene expression→*GRIN2A* → *CAPN2* → *MAPT*→AD as the fifth. Under the MCC between-node correlation, the PIS for these top 5 pathways are 13.80, 12.32, 8.87, 8.84 and 7.42 respectively. The top 2 pathways have comparable PIS, which are much higher than that of other pathways. Thus, the top 2 pathways are likely essential for AD development and can be chosen for further experimental verifications. The detailed results for the rank of the total 33 pathways are presented in the Table S1.

**Table 2:** The top 5 pathways identified by each method from APOE genotype to AD in ROSMAP study

Method	Order				
	1	2	3	4	5
Pearson-SP	*Path*_1_ (152.8)	*Path*_2_ (69.90)	*Path*_3_ (69.01)	*Path*_4_ (59.54)	*Path*_5_ (32.35)
Spearman-SP	*Path*_1_ (246.54)	*Path*_2_ (154.98)	*Path*_4_ (125.08)	*Path*_5_ (96.00)	*Path*_3_ (66.61)
DC-SP	*Path*_1_ (57.84)	*Path*_3_ (41.32)	*Path*_2_ (35.91)	*Path*_4_ (31.42)	*Path*_6_ (24.32)
MIC-SP	*Path*_3_ (3063.17)	*Path*_1_ (1136.26)	*Path*_6_ (1099.96)	*Path*_2_ (743.01)	*Path*_4_ (380.01)
MI-SP	*Path*_3_ (60.95)	*Path*_6_ (39.72)	*Path*_2_ (33.01)	*Path*_5_ (25.26)	*Path*_1_ (21.32)
MCC-SP	*Path*_1_ (13.80)	*Path*_3_ (12.32)	*Path*_4_ (8.87)	*Path*_6_ (8.84)	*Path*_2_ (7.42)

Note: The pathway importance scores (PISs) were shown in the parenthesis. Note that the PIS is only comparable across one specific method

***Path***_**1**_: *APOE* genotype →*APOE* gene expression→*GRIN2A* → *CAPN2* → *MAPT*→AD

***Path***_**2**_: *APOE* genotype →*APOE* gene expression →*GRIN2A* → *MAPK1* → *CASP3* → AD

***Path***_**3**_: *APOE* genotype →*APOE* gene expression →*GRIN2A* → *NOS1* → AD

***Path***_**4**_: *APOE* genotype →*APOE* gene expression →*CACNA1C* → *CAPN2* → *MAPT*→AD

***Path***_**5**_: *APOE* genotype →*APOE* gene expression→*CACNA1C* → *MAPK1* → *CASP3* → AD

***Path***_**6**_: *APOE* genotype →*APOE* gene expression→*CACNA1C* → *NOS1* → AD

## Discussion

Most disease-associated genetic variants lie in the noncoding regions of the genome and gene expression levels can bridge the gap between genetic variant and disease. Often, there are multiple pathways from the genetic variant to the final disease, we have developed a powerful integration method, MCC-SP, to rank and identify these multiple potential pathways. Various simulations under different sample size and different between-node correlation pattern have shown that the proposed method has better performance than other competing methods. ROSMAP data analysis illustrates that the method can partially detect the mechanism from *APOE* genotype to AD through gene expression enriched in AD pathway. The term “integration” here can be interpreted that we have integrated the suitable between-node correlation with the K shortest paths algorithm in graph theory. MCC-SP is essentially network-based and has different model assumptions from traditional TWAS. Statistically, it will lose efficiency if we know the network structure while ignore it during the inference. In this sense, MCC-SP provided an alternative and complement from TWAS to dissect the pathway mediating the genetic variant and the complex disease. MCC-SP are not only limited to gene expression but can be extended to other molecular phenotypes (e.g. proteomics). Note that the relationship among genes may be a mixture of many possible so called “correlations” rather than endorsed to one of the six suggested functions only. Currently, it is hard to extend MCC-SP to summary-level data. For summary level data, one key step is to calculate the correlation matrix among the genes and the traits. If the correlation is linear, we can implement this using some well-known methods [35, 36]. However, as we show here, various nonlinear relationships exist and it is hard to calculate the complex non-linear correlation matrix using summary-level data.

ROSMAP data analysis has found that *APOE* genotype is significant associate with AD (*OR* = 2.8849, *P* = < 0.0001), it is reasonable to utilize gene expression enriched in AD disease pathway to rank and identify the potential pathway from *APOE* to AD. We chose the overlapped genes between the ROSMAP data and those located on the AD disease pathway into the analysis. The top 2 pathways have comparable and much higher pathway important scores (PIS) than other pathways, which indicates that these two pathways may play important roles in AD development. The most important pathway identified is *APOE* genotype →*APOE* gene expression→*GRIN2A* → *CAPN2* → *MAPT*→AD. The *APOE* genotype can regulate its gene expression, in the central nervous system (CNS), *LDLR* family is intimately involved in neuronal signal transduction, modulation of ligand-gated ion channels, and regulating neurite outgrowth, synapse formation and neuronal migration. *ApoE* binds to the highly conserved low-density lipoprotein receptor *(LDLR*) family [37] including *LRP1* and *ApoER2*, while *ApoER2* was reported to bind *NMDAR* (*GRIN2A* belongs to this family) [38, 39]. The *NMDAR* is a cation channel highly permeable to calcium and plays critical roles in governing normal and pathologic functions in neurons. Calcium entry through *NMDAR* can lead to the activation of the Ca2 + −dependent protease, calpain [39, 40]. Gene *MAPT* belongs to the family of *Tau* and *CAPN2* belongs to the family of *Calpain. Calpain*-mediated tau cleavage can play an important role under neurodegenerative conditions [38, 41]. It has been shown that calpain activation results in the generation of several N-terminal tau fragments, which can be detected in mitochondria present in synaptosomal fractions obtained from AD brains [38, 41, 42]. In addition, overexpression of *NMDAR2B* in an inflammatory model of Alzheimer’s disease, which can be modulated by *NOS* (*NOS1* belongs to this family) inhibitors [43]. Further independent sample validation and experimental study can be conducted to validate these findings.

One limitation of our method is that we assume the network or pathway structure is assumed to be known (e.g. AD pathway in our ROSMAP data analysis). Little attention has been paid on the network structure learning problem, which means determining every between-node link with highest degree of data matching, and often one joint distribution of variables can reflect more than one network structure. Actually, most biologists and clinical researchers usually have some prior on the interplay between the biological components and can depict more or less the specific network or pathway for the corresponding biological process. Meanwhile, numerous databases (e.g. KEGG) can be further borrowed to establish the network structure. Even so, MCC-SP is unable to deal with the loop network. Another limitation is that there is lack of test for the significance of the pinpointed pathway, for example, the proposed method is unable to test whether the order of identified pathways is significant or not. Some nonparametric techniques (e.g. permutation and bootstrap) may be further developed to solve such problems. In practice, once we have obtained the rank of the pathways, one key question is which pathway should be selected for further experimental verification. Here we have provided the PIS to quantify the importance of each pathway, we use *q*_50_ as the threshold to indicate that those pathways with effect greater than the median value, will have the PIS greater than one. Regardless of the threshold, PIS is essentially the product of the “correlation” along the specific pathway. Indeed, even for two pathways with similar PIS, MCC-SP still give the different ranks. For example, in our real data analysis, the PIS for *Path*_3_ and *Path*_4_ are quite close (8.87 vs 8.84). This indicates that these two pathways may have equivalent effect and importance but with different ranks. In practice, we recommend choosing those pathways having comparable PIS with the top one for further experimental verification in a cost-effective manner.

## Conclusions

We proposed an integration method called MCC-SP, identifying the causal pathway effect within a network from genetic variant to the disease. MCC-SP is effective and powerful at identifying the specific pathways contributing that cause disease, and can rank these potential pathways, so it can provide new insights into underlying mechanisms and can provide a more comprehensive approach to studying the effects of specific pathways on disease.

## Method

### Six between-node correlation measures

#### Pearson correlation coefficient

The Pearson correlation coefficient is used as an indicator to measure the strength of linear correlation between two random variables X and Y.

$$ r=\frac{\sum \left(X-\overline{X}\right)\left(Y-\overline{Y}\right)}{\sqrt{\sum {\left(X-\overline{X}\right)}^2\sum {\left(Y-\overline{Y}\right)}^2}}, $$

where $\overline{X},\overline{Y}$ represent the mean of the two variables respectively. The range of *r* is [−1, 1]. When *r* = 0, there is no linear relationship between the two variables.

#### Spearman correlation coefficient

The Spearman correlation coefficient indicates the degree of monotonic correlation between two variables *X* and *Y*, which is essentially a linear correlation of the ranks of *X* and *Y*. It is free of the distribution of variables and is defined as follows:

$$ \rho =\frac{\sum_{i=1}^n\left({r}_i-\overline{r}\right)\left({s}_i-\overline{s}\right)}{\sqrt{\sum_{i=1}^n{\left({r}_i-\overline{r}\right)}^2}\sqrt{\sum_{i=1}^n{\left({s}_i-\overline{s}\right)}^2}}, $$

where *r*_*i*_ (*s*_*i*_) represents the rank of *x*_*i*_ (*y*_*i*_) in sample *X* (*Y)*, and the range of *ρ* is [−1, 1]. When *ρ* = 0, there is no monotonic relationship between the two variables. When *ρ* > 0, the relationship between the two variables increases monotonically; when *ρ* < 0, it decreases monotonically.

#### Distance correlation

The distance-related *R*(*X*, *Y*) is different from the previous correlations based on the covariance matrix and variance matrix. It measures the correlation between variables by calculating the Euclidean distance of the sample itself. *R*(*X*, *Y*) is non-negative and can be used to measure the correlation between *X* and *Y* with any dimension. *R*(*X*, *Y*) = 0 indicates that *X* and *Y* are independent.

Suppose the sample data is (*X*, *Y*) = {(*X*_*k*_, *Y*_*k*_) : *k* = 1, …, *n*}, and define the following quantities

$$ {a}_{kl}={\left|{X}_k-{X}_l\right|}_p,\kern0.5em {\overline{a}}_{k.}=\frac{1}{n}{\sum}_{l=1}^n{a}_{kl},\kern0.5em {\overline{a}}_{.l}=\frac{1}{n}{\sum}_{k=1}^n{a}_{kl},\kern0.5em {\overline{a}}_{..}=\frac{1}{n^2}{\sum}_{k,l=1}^n{a}_{kl},{A}_{kl}={a}_{kl}-{\overline{a}}_{k.}-{\overline{a}}_{.l}+{\overline{a}}_{..}. $$

$$ {b}_{kl}={\left|{Y}_k-{Y}_l\right|}_q,\kern0.5em {B}_{kl}={b}_{kl}-{\overline{b}}_{k.}-{\overline{b}}_{.l}+{\overline{b}}_{..},\kern0.5em k,l=1,2,\dots, n. $$

The empirical distance covariance is defined as

$$ {V}_n^2\left(X,Y\right)=\frac{1}{n^2}{\sum}_{k,l=1}^n{A}_{kl}{B}_{kl}, $$

Similarly, *V*_*n*_(*X*) is defined by

$$ {V}_n^2(X)={V}_n^2\left(X,X\right)=\frac{1}{n^2}{\sum}_{k,l=1}^n{A}_{kl}^2. $$

The empirical distance correlation coefficient *R*_*n*_(*X*, *Y*) is defined as:

$$ {R}_n\left(X,Y\right)=\sqrt{R_n^2\left(X,Y\right)}=\left\{\begin{array}{c}\sqrt{\frac{V_n^2\left(X,Y\right)}{\sqrt{V_n^2(X){V}_n^2(Y)}}},\kern1em {V}_n^2(X){V}_n^2(Y)>0\\ {}0\kern3.75em {V}_n^2(X){V}_n^2(Y)=0\end{array}\right.. $$

#### Mutual information based on kernel density estimation (MI)

Mutual Information is a useful measure of information in information theory. It can be seen as the amount of information about a random variable contained in a random variable, or a random variable due to the knowledge of another random variable. Mutual Information does not need to make any assumption about the nature of the relationship between variable characteristics and is defined as

$$ I\left(X,Y\right)={\int}_Y{\int}_Xp\left(x,y\right)\mathit{\log}\left(\frac{p\left(x,y\right)}{p(x)p(y)}\right) dxdy, $$

where *p*(*x*, *y*) is the joint probability density function of (*X*, *Y*), and *p*(*x*), *p*(*y*) are the corresponding marginal density functions of (*X*, *Y*), respectively. *I*(*X*, *Y*) can be viewed as the expected value of $\log \left(\frac{p\left(x,y\right)}{p(x)p(y)}\right)$ (Point Mutual Information, PMI), which is

$$ I\left(X,Y\right)=E\left(\log \left(\frac{\mathrm{p}\left(x,y\right)}{p(x)p(y)}\right)\right). $$

The value of mutual information can also be expressed as Kullback–Leibler divergence (also known as relative entropy),

$$ I\left(X,Y\right)=H(Y)-H\left(Y|X\right), $$

where *H*(*Y*) is the entropy of *Y*, referring to the uncertainty of *Y*, and *H*(*Y*| *X*) is the uncertainty of *Y* given *X*. Thus, *I*(*X*, *Y*) can be interpreted as a quantity introduced by *X* to reduce the uncertainty of *Y*. Therefore, the closer the relationship between *X* and *Y*, the greater the *I*(*X*, *Y*). *I*(*X*, *Y*) = 0 when two variables are independent.

The calculation of MI requires the estimation of the density functions, we adopt the kernel density estimation method. For the one-dimension marginal density, we assume that the data *x*_1_, *x*_2_, …, *x*_*n*_, are taken from the continuous distribution *p*(*x*). A kernel density estimate is defined as

$$ \hat{p}(x)=\frac{1}{nh}{\sum}_{i=1}^n{\omega}_i=\frac{1}{nh}{\sum}_{i=1}^nK\left(\frac{x-{x}_i}{h}\right), $$

where *h* is the bandwidth and *K*(∙) is the kernel function, *K*(*x*) > 0, ∫*K*(*x*)*dx* = 1. At present study, we chose the commonly used *Gauss* kernel function.

For the two-dimension joint density, assuming that the data *X*, *Y* be a bivariate sample drawn from a common distribution described by the density function. The bivariate kernel density estimation can be defined to be

$$ {\hat{f}}_H\left(\boldsymbol{z};\boldsymbol{H}\right)=\frac{1}{n}{\sum}_{i=1}^n{K}_H\left(\boldsymbol{z}-{\boldsymbol{Z}}_{\boldsymbol{i}}\right), $$

where =(***x***, ***y***)^*T*^ ***Z***_***i***_ = (***X***_***i*1**_, ***Y***_***i*2**_)^*T*^, *i* = 1, 2, …, *n*.

Here ***H*** is the bandwidth (or smoothing) 2 × 2 matrix which is symmetric and positive definite; *K*(∙) is the bivariate kernel function which is a symmetric multivariate density and ${K}_H\left(\boldsymbol{z}\right)={\left|\boldsymbol{H}\right|}^{-\frac{1}{2}}K\left({\boldsymbol{H}}^{-\frac{1}{2}}\boldsymbol{z}\right)$. At present study, we use the standard multivariate normal kernel: ${K}_H\left(\boldsymbol{z}\right)={\left(2\pi \right)}^{-\frac{d}{2}}{\left|\boldsymbol{H}\right|}^{-\frac{d}{2}}\exp \left(-\frac{1}{2}{\boldsymbol{z}}^T{H}^{-1}\boldsymbol{z}\right)$ with d = 2.

### Maximal information coefficient (MIC)

The idea of the MIC is that if there is a relationship between two variables, one can draw a grid on the scatter plot of the two variables, which partitions the data to encapsulate that relationship. To calculate the MIC of a set of two variables, we explore all grids up to a maximal grid resolution which is dependent on the sample size, computing for each pair of integers (*X*, *Y*) the largest possible mutual information achievable by any *X* -by- *Y* grid applied to the data [44]. These mutual information values are normalized such that the grids of different dimensions are comparable (values between 0 and 1). We define the characteristic matrix M (*M* = (*m*_*X*, *Y*_)), where *m*_*X*, *Y*_ is the largest normalized mutual information value in all grids, and MIC is the largest value in M. Given the current data set D, the largest mutual information value is recorded as *I*(*D*, *X*, *Y*), which can be further standardized as

$$ \mathrm{M}{(D)}_{x,y}=\frac{I\left(D,x,y\right)}{\log \left(\mathit{\min}\left\{x,y\right\}\right)}, $$

M(*D*)_*x*, *y*_ ranges between 0 and 1.

Suppose that the sample size is *n*, and the number of grid divisions is less than *B*(*n*). Then the maximal information coefficient (MIC) is defined as

$$ \mathrm{MIC}\left(\mathrm{D}\right)={\max}_{xy<B(n)\left\{M{(D)}_{x,y}\right\}}. $$

The MIC is a generalized correlation and ranges between 0 and 1. It can detect a wide range of correlations when the sample size is sufficiently large. When MIC = 0, *X* and *Y* are independent.

### Maximal correlation coefficient (MCC)

MCC first performs the best conversion on two random variables *X* and *Y*, and then uses the Pearson correlation coefficient to calculate the correlation. The best transform estimation is based only on the data samples and has minimal assumptions about the data allocation and the form of the best transform. In particular, we don’t need the transformation functions to come from a particular parameterized family or even monotonic. Let *X*, *Y* be random variables defined in the probability space (*X*, *A*, *P*) and they are randomly selected at (*X*, *B*_1_) and (*Y*, *B*_2_). Map *X* : (*X*, *A*) → (*X*, *B*_1_), *Y* : (*Y*, *A*) → (*Y*, *B*_2_) generates a subalgebra *A*_1_ = *X*^−1^(*B*_1_) and *A*_2_ = *Y*^−1^(*B*_2_) in *A*. *P*_*i*_ is a measure of *P* on *A*, *i* = 1, 2. Let function *φ* have a finite second moment *E*|*φ*|^2^ =  ∫ |*φ*|^2^*dP* < ∞, and have an inner product (*φ*_1_, *φ*_2_) = *E*(*φ*_1_, *φ*_2_), and *L*^2^ = *L*^2^(*P*) is the Hilbert space of *A*-measurable function *φ*; ${L}_i^2={L}_i^2(P)$ is the Hilbert space of *A*_*i*_ measurable function *φ* with finite second moment and same inner product. Define MCC between *X* and *Y* as: MCC(*X*_1_, *X*_2_) = sup {*ρ*(*φ*_1_(*X*_1_), *φ*_2_(*X*_2_))}, where *ρ*(∙) is the Pearson correlation coefficient, and ${\varphi}_1(X)\in {L}_1^2$, ${\varphi}_2(Y)\in {L}_2^2$.

In principle, MCC measures the cosine of the angle between the linear subspaces of mean zero square integrable real-valued random variables. The maximal correlation is the supremum of *ρ*(*φ*_1_(*X*), *φ*_2_(*Y*)). The key is to choose the right function to get the exact value of the upper bound. Two variables are independent, *MCC*(*X*, *Y*) = 0, is equivalent to that *L*^2^’ s two subspaces ${L}_i^2$ (*i* = 1, 2) are orthogonal. If the relationship between *X* and *Y* is linear, the MCC will degenerate into the Pearson correlation coefficient. At present study, we calculate the MCC by the commonly used ACE method, which can be easily obtained by R package *acepack* [45].

### K shortest paths algorithm

In a directed network, K Shortest Paths Algorithm is used to find the path with the smallest weight from one starting node to the end node. Here the starting node is a genetic variant (e.g. SNP) and the end node is complex disease (e.g. AD). Given that the goal is to find the pathway with relatively large effect, the first step is to transform the between-node correlation such that we can apply the K Shortest Paths Algorithm. Let *r*_*ij*_ represent the general correlation (e.g. one of the above correlation quantities) between the two nodes *M*_*i*_ and *M*_*j*_, and the direction is *X*_*i*_ → *X*_*j*_. Suppose there is a simple path from the SNP *X* to the outcome disease *Y*, *X* → *M*_1_ → *M*_2_ → *Y*. Then, along this pathway, the effect of *X* on *Y* can be represented as ${r}_{X,{M}_1}\times {r}_{M_1,{M}_2}\times {r}_{M_2,Y}$. We transform *r*_*ij*_ by the following equation:

$$ {r_{ij}}^{\prime }=\log \frac{1}{r_{ij}}, $$

where *i*, *j* represents nodes such as *X*, *Y*, and *M*_*i*_ (*i* = 1, 2), then the weight along this pathway is

$$ {r_{X,{M}_1}}^{\prime }+{r_{M_1,{M}_2}}^{\prime }+{r_{M_2,Y}}^{\prime }=\log \frac{1}{r_{X,{M}_1}}+\log \frac{1}{r_{M_1,{M}_2}}+\log \frac{1}{r_{M_2,Y}}=\log \frac{1}{r_{X,{M}_1}\times {r}_{M_1,{M}_2}\times {r}_{M_2,Y}}. $$

Such simple reciprocal function transforms the maximum value of the weight into its minimum, and the log transform converts the product to the summation, then the K Shortest Paths Algorithm can be easily implemented by the commonly used deviation path algorithm [34].

### Definition of the pathway importance score (PIS)

It naturally defined the pathway effect to be the product of between-node connection along specific pathway, for instance, the effect of the simple pathway *X* → *M*_1_ → *M*_2_ → *Y* is defined to be ${r}_{X,{M}_1}\times {r}_{M_1,{M}_2}\times {r}_{M_2,Y}$. Suppose that there are totally Q pathways within one network (e.g. there are 33 pathways from APOE genotype to AD mediated by gene expression in our real data analysis), we denote *q*_50_ to be the median of effects of all these Q pathways, then the pathway importance score (PIS) for the *i* th pathway is

$$ {PIS}_i=\left( the\ effect\ of\  ith\  pathway\right)/{q}_{50}, $$

where *i* = 1, …Q. Intuitively, for the pathway with effect greater than the median value, it should be relatively important and the PIS should be greater than one, otherwise it should be less than one. PIS provides a simple way to quantify the importance of pathway from the genetic variant to the outcome. In practice, once obtaining the rank of all pathways, one can chose those having comparable PIS with the top one for further experimental verification.

### Simulation

**Fig. 3:**
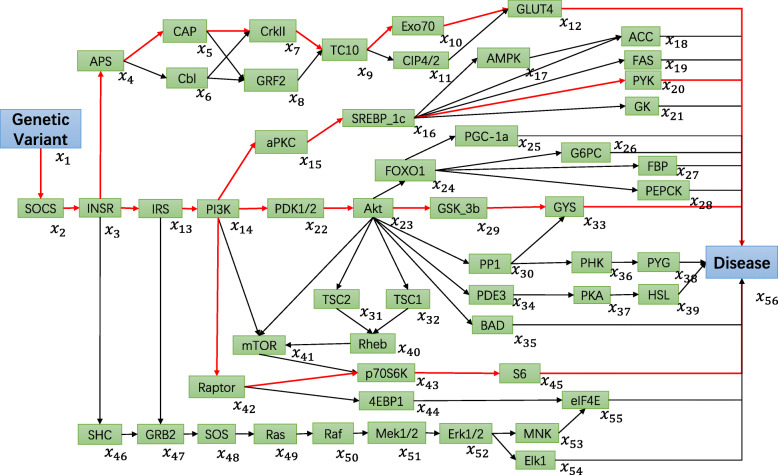
The simulated network from genetic variant to disease constructed from the insulin signaling pathway from KEGG. The hypothesis is that the genetic variant can affect the disease through the gene expression on the multiple pathways. The genes and the direction are highlighted as the green box and the black arrow. The 23 edges included in the 4 effective pathways are highlighted in red

Various simulations were conducted to assess the performance of the above six correlation measurements together with the K Shortest paths algorithm, in identifying the potential pathway from genetic variant to the disease outcome. To make the simulations more realistic, we designed the network (Fig. 3) based on the insulin signaling pathway from KEGG, we simulated that the genetic variant can affect disease mediated by gene expression enriched on the insulin signaling pathway. We generated starting node *X*_1_ from *N*(0, 1) and then generated the other nodes following the network structure (totally 56 nodes and 82 edges). If the direction for node *X*_*i*_ and *X*_*j*_ is *X*_*i*_ → *X*_*j*_ (*i* = 1, …55, *j* = 2, …56, *i* < *j*), given *X*_*i*_, we generate *X*_*j*_ as *x*_*j*_ = *μ*_*j*_ + *φ*(*x*_*i*_) + *ε*_*j*_, where *μ*_*j*_ is the intercept and *ε*_*j*_ is the error term following $N\left(0,{\sigma}_j^2\right)$, the parameter *μ*_*j*_ and *σ*_*j*_ can be assigned to ensure the mean and variance of node *X*_*j*_ to be zero and unit. The function *φ* is pre-specified based on the designed relationship between *X*_*i*_ and *X*_*j*_, for instance, $\varphi \left({x}_i\right)=\sqrt{C-{x}^2}$, *φ*(*x*_*i*_) = *x*_*i*_, $\varphi \left({x}_i\right)={x}_i^2$, *φ*(*x*_*i*_) = cos(*x*_*i*_) and *φ*(*x*_*i*_) = sin(2*x*_*i*_) for the arcuate, linear, quadratic, cosine and sine relationship, respectively. The correlation strength between these two nodes can be measured as the linear correlation coefficient between *φ*(*x*_*i*_) and *x*_*j*_, *corr*(*φ*(*x*_*i*_), *x*_*j*_)(*i* = 1, 2, …, 56), which is used as the weight between these two nodes. For instance, suppose that there is the quadratic relationship between *X*_1_ and *X*_2_, then *X*_2_ can be generated as ${x}_2={\mu}_2+{\beta}_{12}{x}_1^2+{\varepsilon}_2$, where *β*_12_ is the pre-specified parameter, *ε*_2_ is the error term following $N\left(0,{\sigma}_2^2\right)$. We set ${\sigma}_2=\sqrt{1-2{\beta}_{12}^2}$ and *μ*_2_ =  − *β*_12_. If the downstream *x*_*j*_ is generated from multiple upstream nodes, similar designs can be conducted. For instance, the node *x*_9_ is linearly dependent on both *x*_7_ and *x*_8_, then *x*_9_ = *μ*_*j*_ + *β*_79_*x*_7_ + *β*_89_*x*_8_ + *ε*_9_, and we also ensured the mean and variance of *x*_9_ to be zero and unit respectively.

The goal is to determine the suitable correlation measure that can capture the between-node relationship in the network and can correctly pinpoint the top few pathways with large effects using the K shortest paths algorithm. Here, we assigned 4 pathways with relatively large effect, and these 4 pathways covered 23 edges (Fig. 3). Note that here we chose the correlation strength between nodes on these 4 pathways randomly from *unif*(0.75,1) and that on the other pathways randomly from *unif*(0,0.25), to make these 4 pathways have relatively larger effects than other pathways. Two scenarios are considered as follows: (1) all the between-node correlations are linear, and (2) among the 23 edges, we randomly set the proportion of the nonlinear edge (e.g. the between-node correlation is nonlinear) to be 30, 40, 50 and 60%, respectively (See Figures S16, S17, S18, S19 for details). The nonlinear relationship (*φ*(∙)) includes *x*^2^, cos(*x*), sin(2*x*), $\sqrt{C-{x}^2}$. Here we used *φ*(*x*_*i*_) = sin(2*x*_*i*_) + *ε* as the sine relationship, given that the sine function is close to linear in the interval [−*π*, *π*]. We set the proportion of nonlinear relationship to be 30% (5 edges having cosine and 2 edges having quadratic relationship), 40% (6 edges having cosine and 3 edges having quadratic relationship), 50% (8 edges having cosine and 4 edges having quadratic relationship) and 60% (8 edges having cosine and 5 edges having quadratic and 1 edge having $\sqrt{C-{x}^2}$ relationship). We kept the edges with the above nonlinear correlation pattern to be the same in each replicate for better comparison. For each simulation setting, we first generated the whole population with sample size 50,000, then we calculate the 82 between-node correlations (i.e. linear or nonlinear correlation between the two neighbored nodes) to derive and obtain the true order of the effects of the pathways. We randomly chose the 100, 300, 500 samples without replacement from 50,000 population, and replicated 500 simulations for each scenario.

We aimed to assess the performance of the methods of the existing six correlation metrics with K shortest paths algorithm, which can be labeled as Person-SP, Spearman-SP, DC-SP, MI-SP, MIC-SP, MCC-SP. Here we preferred two criteria to evaluate the ability of the six integration methods to pinpoint the pathways with relatively large effect. The two criteria are 1) all-right: it is the most stringent and means that the top 4 pathways can be precisely allocated with the same order as their effects from large to small; the more times to find the top 4 pathways, the better the method; and 2) range-right: it means that the top 4 pathways with some effects are ranked in top 4, while the order can be allowed to be chaotic. For instance, if the top 4 paths are sorted as *Path*_1_, *Path*_2_, *Path*_3_ and *Path*_4_ based on the pathway effect from large to small. The “all-right” criteria means that the top 4 paths must be *Path*_1_, *Path*_2_, *Path*_3_, *Path*_4_, while the “range-right” criteria means the top 4 paths just include *Path*_1_, *Path*_2_, *Path*_3_, *Path*_4_, regardless of the order. For example, it can be *Path*_2_, *Path*_3_, *Path*_4_, *Path*_1_ or any other order patterns.

### Application datasets

We applied these six integration methods to identify the potential causal pathway from *APOE* genotype to AD, with the network constructed from KEGG-based Alzheimer’s disease pathway (Figure S20). The Religious Orders Study and Memory and Aging Project (ROSMAP) Study is divided into two parts, ROS (The Religious Orders Study) and The Memory and Aging Project (MAP). Details about the ROSMAP can be found in previous studies [46, 47] and the website https://www.synapse.org/#!Synapse:syn3219045. In ROSMAP, Alzheimer’s Disease status was determined by a computer algorithm based on cognitive test performance with a series of discrete clinical judgments made in series by a neuropsychologist and a clinician.

DNA has been used to characterize apolipoprotein E allele status (*APOE*), and more recently, it has been used to generate genome-wide genotyping data generated on a Affymetrix 6.0 platform and imputed to 2.2 million single nucleotide polymorphisms (SNPs) with HapMAP [46, 47]. The *APOE* genotype is defined as a 0–1 variable. Following previous studies [48–50], if one or both genotypes are ε4, it is assigned to be 1, otherwise it is set to be 0. The gray matter of the dorsolateral prefrontal cortex of the subject was used to extract RNA from the ROS and MAP cohorts. Agilent Bioanalyzer performs a quality assessment of samples quantified by Nanodrop. The strand-specific dUTP method [51] with poly-A selection [52] was used in the Genomics platform of Broad Institutes for the preparation of RNA-Seq libraries. The quality of the RNA-Seq sample (Bioanalyzer RNA Integrity (RIN) score > 5) and the number threshold (5 μg) requirements were required. Sequencing was performed on the Illumina HiSeq. Before applying RSEM to estimate the expression levels of all transcripts, the non-gapped aligner Bowtie was used to compare the reads to the transcriptome reference. The result of the data RNA-Seq pipeline is the FPKM value. The quantile normalization method will first be applied to FPKM and subsequently used to eliminate potential batch effect using “*combat*” package.

**Fig. 4:**
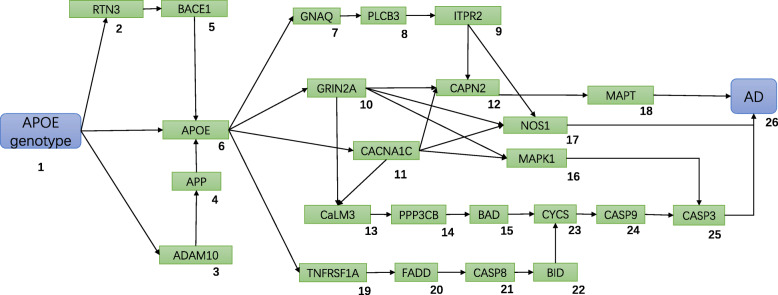
The whole network from APOE genotype to AD constructed from KEGG-based Alzheimer’s disease pathway. The hypothesis is that the APOE genetic variant can affect AD through the gene expression on Alzheimer’s disease pathway. Multiple pathways with chain structure can be formulated with the staring node APOE genotype and the end node AD. The gene and directions are highlighted with a green frame and a black arrow

The study used 364 samples (236 females and 128 males) from ROSMAP, with 163 from MAP and 201 from ROS. The age of death was between 67 and 90 years. Among these samples, 192 samples had AD and 172 were normal controls. We first performed a simple logistic regression between *APOE* genotype and AD (β = 1.0595, *p* < 0.0001). It is necessary to explore the pathway mechanism behind this association. We mapped all gene expression from ROSMAP to the KEGG Alzheimer’s disease pathway to determine the candidate gene expression. The *APOE* genotype were linked to the three genes *(RTN3*, *ADAM10* and *AOPE*), which located on the left of the Alzheimer’s disease pathway. Then, the other downstream genes are connected following the Alzheimer’s disease pathway structure, and finally connected to AD. There is a total of 24 genes and 26 nodes in the network. The starting node of the whole network is the *APOE* genotype and the terminating node is AD (Fig. 4).

## Supplementary information

**Additional file 1: Table S1.** The rank of the total 33 pathways for 6 integration methods. **Figure S1.** The proportion correctly pinpointing top 4 pathways under sample size 500 and nonlinear proportion 60%. **Figure S2.** The proportion correctly pinpointing top 4 pathways under sample size 500 and nonlinear proportion 30%. **Figure S3.** The proportion correctly pinpointing top 4 pathways under sample size 500 and nonlinear pattern being *φ*(*x*_*i*_) = sin(2*x*_*i*_) + *ε*. **Figure S4.** The proportion correctly pinpointing top 4 pathways under sample size 500 and nonlinear pattern being exponential and reciprocal. **Figure S5. T**he proportion correctly pinpointing top 4 pathways under sample size 500 and nonlinear proportion 40% (including NPR). **Figure S6.** The proportion correctly pinpointing top 4 pathways under sample size 300 and nonlinear proportion 30%. **Figure S7.** The proportion correctly pinpointing top 4 pathways under sample size 300 and nonlinear proportion 40%. **Figure S8.** The proportion correctly pinpointing top 4 pathways under sample size 300 and nonlinear proportion 50%. **Figure S9.** The proportion correctly pinpointing top 4 pathways under sample size 300 and nonlinear proportion 60%. **Figure S10.** The proportion correctly pinpointing top 4 pathways under sample size 300 and nonlinear pattern being *φ*(*x*_*i*_) = sin(2*x*_*i*_) + *ε*. **Figure S11.** The proportion correctly pinpointing top 4 pathways under sample size 100 and nonlinear proportion 30%. **Figure S12.** The proportion correctly pinpointing top 4 pathways under sample size 100 and nonlinear proportion 40%. **Figure S13.** The proportion correctly pinpointing top 4 pathways under sample size 100 and nonlinear proportion 50%. **Figure S14.** The proportion correctly pinpointing top 4 pathways under sample size 100 and nonlinear proportion 60%. **Figure S15.** The proportion correctly pinpointing top 4 pathways under sample size 100 and nonlinear pattern being (*x*_*i*_) = sin(2*x*_*i*_) + *ε* . **Figure S16.** The network when there are 30% nonlinear between-node connection in the 4 effective pathways. **Figure S17.** The network when there are 40% nonlinear between-node connection in the 4 effective pathways. **Figure S18.** The network when there are 50% nonlinear between-node connection in the 4 effective pathways. **Figure S19.** The network when there are 60% nonlinear between-node connection in the 4 effective pathways. **Figure S20.** The Alzheimer’s disease pathway downloaded from KEGG.

## Data Availability

The datasets analyzed for this study can be found in the ROSMAP (https://www.synapse.org/#!Synapse:syn3219045). The code of MCC-SP is freely available at GitHub (https://github.com/zhuyuchen95/ADnet).

## Abbreviations

GWAS: Genome-wide association studies
SNP: Single nucleotide polymorphism
MCC: The maximal correlation coefficient
PIS: The pathway importance score
AD: Alzheimer’s disease
eQTLs: Expression quantitative trait loci
TWAS: Transcriptome-wide association studies
CaMMEL: The causal multivariate mediation within extended linkage disequilibrium
ROSMAP: The Religious Orders Study and the Memory and Aging Project.
MI: Mutual information
MIC: Maximal Information Coefficient
CNS: The central nervous system
LDLR: Low-density lipoprotein receptor
