# Supplementary material for: MCC-SP: a powerful integration method for identification of causal pathways from genetic variants to complex disease
Source: BMC Genet. 2020 Aug 26;21:90. doi: 10.1186/s12863-020-00899-3 (PMC7477886; doi:10.1186/s12863-020-00899-3)
Supplement: Supplementary file 1 — Additional file 1: Table S1. The rank of the total 33 pathways for 6 integration methods. Figure S1. The proportion correctly pinpointing top 4 pathways under sample size 500 and nonlinear proportion 60%. Figure S2. The proportion correctly pinpointing top 4 pathways under sample size 500 and nonlinear proportion 30%. Figure S3. The proportion correctly pinpointing top 4 pathways under sample size 500 and nonlinear pattern being φ(xi) = sin(2xi) + ε. Figure S4. The proportion correctly pinpointing top 4 pathways under sample size 500 and nonlinear pattern being exponential and reciprocal. Figure S5. The proportion correctly pinpointing top 4 pathways under sample size 500 and nonlinear proportion 40% (including NPR). Figure S6. The proportion correctly pinpointing top 4 pathways under sample size 300 and nonlinear proportion 30%. Figure S7. The proportion correctly pinpointing top 4 pathways under sample size 300 and nonlinear proportion 40%. Figure S8. The proportion correctly pinpointing top 4 pathways under sample size 300 and nonlinear proportion 50%. Figure S9. The proportion correctly pinpointing top 4 pathways under sample size 300 and nonlinear proportion 60%. Figure S10. The proportion correctly pinpointing top 4 pathways under sample size 300 and nonlinear pattern being φ(xi) = sin(2xi) + ε. Figure S11. The proportion correctly pinpointing top 4 pathways under sample size 100 and nonlinear proportion 30%. Figure S12. The proportion correctly pinpointing top 4 pathways under sample size 100 and nonlinear proportion 40%. Figure S13. The proportion correctly pinpointing top 4 pathways under sample size 100 and nonlinear proportion 50%. Figure S14. The proportion correctly pinpointing top 4 pathways under sample size 100 and nonlinear proportion 60%. Figure S15. The proportion correctly pinpointing top 4 pathways under sample size 100 and nonlinear pattern being (xi) = sin(2xi) + ε . Figure S16. The network when there are 30% nonlinear between-node connection [file 12863_2020_899_MOESM1_ESM.pdf]

Supplementary information

Supplementary Tables

Table S1. The rank of the total 33 pathways for 6 integration methods.

Order \ Method	Pearson-SP	Spearman-SP	DC-SP	MIC-SP	MI-SP	MCC-SP
1	$Path_1$	$Path_1$	$Path_1$	$Path_3$	$Path_3$	$Path_1$
2	$Path_2$	$Path_2$	$Path_3$	$Path_1$	$Path_6$	$Path_3$
3	$Path_3$	$Path_4$	$Path_2$	$Path_6$	$Path_2$	$Path_4$
4	$Path_4$	$Path_5$	$Path_4$	$Path_2$	$Path_5$	$Path_6$
5	$Path_5$	$Path_3$	$Path_6$	$Path_4$	$Path_1$	$Path_2$
6	$Path_6$	$Path_6$	$Path_5$	$Path_5$	$Path_4$	$Path_5$
7	$Path_7$	$Path_7$	$Path_7$	$Path_9$	$Path_9$	$Path_{11}$
8	$Path_8$	$Path_8$	$Path_9$	$Path_7$	$Path_{15}$	$Path_7$
9	$Path_9$	$Path_{12}$	$Path_8$	$Path_{15}$	$Path_8$	$Path_9$
10	$Path_{10}$	$Path_{10}$	$Path_{10}$	$Path_8$	$Path_{13}$	$Path_{20}$
11	$Path_{11}$	$Path_{13}$	$Path_{15}$	$Path_{10}$	$Path_{18}$	$Path_{10}$
12	$Path_{12}$	$Path_{16}$	$Path_{13}$	$Path_{13}$	$Path_7$	$Path_{15}$
13	$Path_{13}$	$Path_9$	$Path_{11}$	$Path_{18}$	$Path_{20}$	$Path_8$
14	$Path_{14}$	$Path_{14}$	$Path_{14}$	$Path_{20}$	$Path_{22}$	$Path_{13}$
15	$Path_{15}$	$Path_{11}$	$Path_{12}$	$Path_{14}$	$Path_{10}$	$Path_{14}$
16	$Path_{16}$	$Path_{15}$	$Path_{18}$	$Path_{22}$	$Path_{17}$	$Path_{23}$
17	$Path_{17}$	$Path_{17}$	$Path_{17}$	$Path_{11}$	$Path_{11}$	$Path_{18}$
18	$Path_{18}$	$Path_{19}$	$Path_{20}$	$Path_{17}$	$Path_{21}$	$Path_{19}$
19	$Path_{19}$	$Path_{21}$	$Path_{19}$	$Path_{19}$	$Path_{12}$	$Path_{22}$
20	$Path_{20}$	$Path_{18}$	$Path_{16}$	$Path_{21}$	$Path_{14}$	$Path_{17}$
21	$Path_{21}$	$Path_{22}$	$Path_{22}$	$Path_{12}$	$Path_{19}$	$Path_{26}$
22	$Path_{22}$	$Path_{20}$	$Path_{21}$	$Path_{16}$	$Path_{16}$	$Path_{21}$
23	$Path_{23}$	$Path_{24}$	$Path_{23}$	$Path_{26}$	$Path_{26}$	$Path_{27}$
24	$Path_{24}$	$Path_{25}$	$Path_{24}$	$Path_{23}$	$Path_{23}$	$Path_{12}$
25	$Path_{25}$	$Path_{23}$	$Path_{26}$	$Path_{24}$	$Path_{24}$	$Path_{16}$
26	$Path_{26}$	$Path_{28}$	$Path_{25}$	$Path_{31}$	$Path_{31}$	$Path_{31}$
27	$Path_{27}$	$Path_{29}$	$Path_{27}$	$Path_{27}$	$Path_{25}$	$Path_{24}$
28	$Path_{28}$	$Path_{30}$	$Path_{28}$	$Path_{25}$	$Path_{27}$	$Path_{25}$
29	$Path_{29}$	$Path_{27}$	$Path_{31}$	$Path_{28}$	$Path_{28}$	$Path_{28}$
30	$Path_{30}$	$Path_{26}$	$Path_{29}$	$Path_{29}$	$Path_{29}$	$Path_{29}$
31	$Path_{31}$	$Path_{31}$	$Path_{30}$	$Path_{30}$	$Path_{30}$	$Path_{30}$
32	$Path_{32}$	$Path_{32}$	$Path_{32}$	$Path_{32}$	$Path_{32}$	$Path_{32}$
33	$Path_{33}$	$Path_{33}$	$Path_{33}$	$Path_{33}$	$Path_{33}$	$Path_{33}$

Note:

$\boldsymbol{Path_1}$: *APOE* genotype →*APOE* gene expression→*GRIN2A*→*CAPN2*→*MAPT*→AD.

$\boldsymbol{Path_2}$: *APOE* genotype →*APOE* gene expression →*GRIN2A*→*MAPK1*→ *CASP3* →AD.

$\boldsymbol{Path_3}$: *APOE* genotype →*APOE* gene expression →*GRIN2A*→*NOS1*→AD.

$\boldsymbol{Path_4}$: *APOE* genotype →*APOE* gene expression →*CACNA1C*→*CAPN2*→*MAPT*→AD.

$\boldsymbol{Path_5}$: *APOE* genotype →*APOE* gene expression→*CACNA1C*→*MAPK1*→ *CASP3* →AD.

$\boldsymbol{Path_6}$: *APOE* genotype →*APOE* gene expression→*CACNA1C*→*NOS1*→AD.

$\boldsymbol{Path_7}$: *APOE* genotype→*RTN3*→*BACE1*→*APOE* gene expression→*GRIN2A*→*CAPN2*→*MAPT*→AD

$\boldsymbol{Path_8}$: *APOE* genotype→*RTN3*→*BACE1*→*APOE* gene expression→*GRIN2A*→*MAPK1*→ *CASP3* →AD

$\boldsymbol{Path_9}$: *APOE* genotype→*RTN3*→*BACE1*→*APOE* gene expression→*GRIN2A*→*NOS1*→AD

$\boldsymbol{Path_{10}}$: *APOE* genotype→*RTN3*→*BACE1*→*APOE* gene expression→*CACNA1C*→*CAPN2*→*MAPT*→AD

$\boldsymbol{Path_{11}}$: *APOE* genotype →*APOE* gene expression→*GNAQ*→*PLCB3*→*ITPR2*→*CAPN2*→*MAPT*→AD

$\boldsymbol{Path_{12}}$: *APOE* genotype →*APOE* gene expression→ *GRIN2A*→*CALM3*→*PPP3CB*→*BAD*→*CYCS*→*CASP9*→ *CASP3*→ AD

$\boldsymbol{Path_{13}}$: *APOE* genotype→*RTN3*→*BACE1*→*APOE* gene expression→*CACNA1C*→*MAPK1*→ *CASP3* →AD

$\boldsymbol{Path_{14}}$: *APOE* genotype→*ADAM10*→*APP*→*APOE* gene expression→*GRIN2A*→*CAPN2*→*MAPT*→AD

$\boldsymbol{Path_{15}}$: *APOE* genotype→*RTN3*→*BACE1*→*APOE* gene expression→*CACNA1C*→*NOS1*→AD

$\boldsymbol{Path_{16}}$: *APOE* genotype →*APOE* gene expression →*CACNA1C*→*CALM3* →*PPP3CB*→*BAD*→*CYCS*→ *CASP9*→*CASP3*→ AD

$\boldsymbol{Path_{17}}$: *APOE* genotype→*ADAM10*→*APP*→*APOE* gene expression→*GRIN2A*→*MAPK1*→ *CASP3* →AD

$\boldsymbol{Path_{18}}$: *APOE* genotype→*ADAM10*→*APP*→*APOE* gene expression→*GRIN2A*→*NOS1*→AD

$\boldsymbol{Path_{19}}$: *APOE* genotype→*ADAM10*→*APP*→*APOE* gene expression→*CACNA1C*→*CAPN2*→*MAPT*→AD

$\boldsymbol{Path_{20}}$: *APOE* genotype →*APOE* gene expression→*GNAQ*→*PLCB3*→*ITPR2*→*NOS1*→AD

$\boldsymbol{Path_{21}}$: *APOE* genotype→*ADAM10*→*APP*→*APOE* gene expression→*CACNA1C*→*MAPK1*→ *CASP3* →AD

$\boldsymbol{Path_{22}}$: *APOE* genotype→*ADAM10*→*APP*→*APOE* gene expression→*CACNA1C*→*NOS1*→AD

$\boldsymbol{Path_{23}}$: *APOE* genotype→*RTN3*→*BACE1*→*APOE* gene expression→*GNAQ*→*PLCB3*→*ITPR2*→*CAPN2* →*MAPT*→AD

$\boldsymbol{Path_{24}}$: *APOE* genotype→*RTN3*→*BACE1*→*APOE* gene expression→*GRIN2A*→*CALM3*→ *PPP3CB*→ *BAD*→*CYCS*→ *CASP9*→*CASP3*→AD

$\boldsymbol{Path_{25}}$: *APOE* genotype→*RTN3*→*BACE1*→*APOE* gene expression→*CACNA1C*→*CALM3*→ *PPP3CB*→*BAD*→*CYCS*→ *CASP9*→*CASP3*→AD

$\boldsymbol{Path_{26}}$: *APOE* genotype→*RTN3*→*BACE1*→*APOE* gene expression→*GNAQ*→*PLCB3*→*ITPR2*→*NOS1*→AD

$\boldsymbol{Path_{27}}$: *APOE* genotype→*ADAM10*→*APP*→*APOE* gene expression→*GNAQ*→*PLCB3*→*ITPR2* →*CAPN2*→ *MAPT*→ AD

$\boldsymbol{Path_{28}}$: *APOE* genotype→*ADAM10*→*APP*→*APOE* gene expression→*GRIN2A*→*CALM3*→*PPP3CB*→*BAD* →*CYCS*→ *CASP9*→*CASP3*→AD

$\boldsymbol{Path_{29}}$: *APOE* genotype→*ADAM10*→*APP*→*APOE* gene expression→*CACNA1C*→*CALM3*→*PPP3CB*→*BAD*→*CYCS*→ *CASP9*→*CASP3*→AD

$\boldsymbol{Path_{30}}$: *APOE* genotype →*APOE* gene expression→*TNFRSF1A*→*FADD*→*CASP8*→*BID*→*CYCS*→*CASP9*→ *CASP3*→AD

$\boldsymbol{Path_{31}}$: *APOE* genotype→*ADAM10*→*APP*→*APOE* gene expression→*GNAQ*→*PLCB3*→*ITPR2*→*NOS1*→AD

$\boldsymbol{Path_{32}}$: *APOE* genotype→*RTN3*→*BACE1*→*APOE* gene expression→*TNFRSF1A*→*FADD*→*CASP8*→*BID*→ *CYCS*→*CASP9*→*CASP3*→AD

$\boldsymbol{Path_{33}}$: *APOE* genotype→*ADAM10*→*APP*→*APOE* gene expression→*TNFRSF1A*→*FADD*→*CASP8*→*BID*→ *CYCS*→*CASP9*→*CASP3*→AD

Supplementary Figures

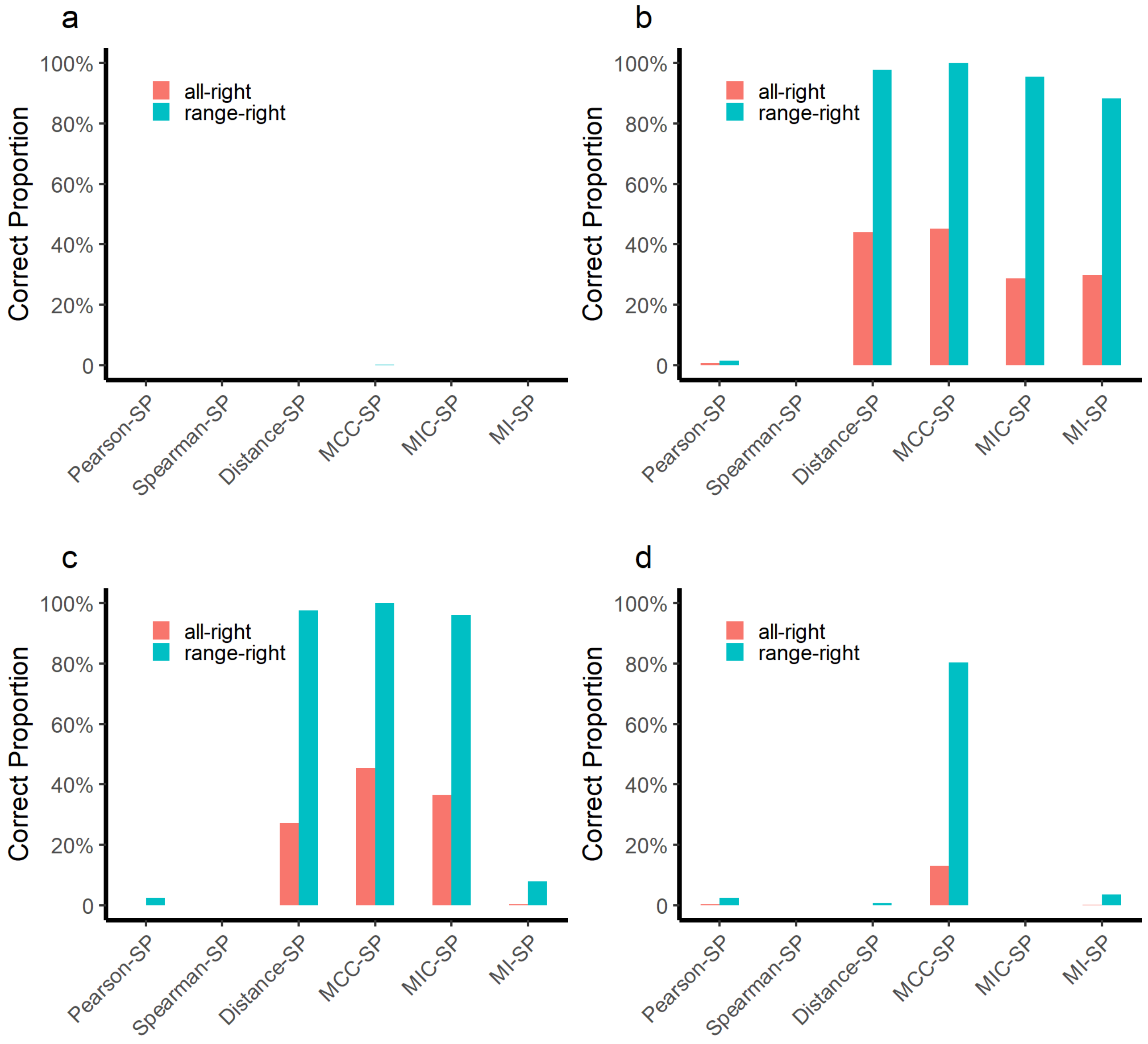

Figure S1. The proportion correctly pinpointing top 4 pathways under sample size 500 and nonlinear proportion 60%. The nonlinear pattern is **(a)** $\varphi(x_i) = \sqrt{C - x_i^2} + \varepsilon$, **(b)** $\varphi(x_i) = \cos(x_i) + \varepsilon$, **(c)** mixed nonlinear pattern (8 edges having cosine and 5 edges having quadratic and 1 edge having arcuate relationship) and **(d)** $\varphi(x_i) = x_i^2 + \varepsilon$ respectively.

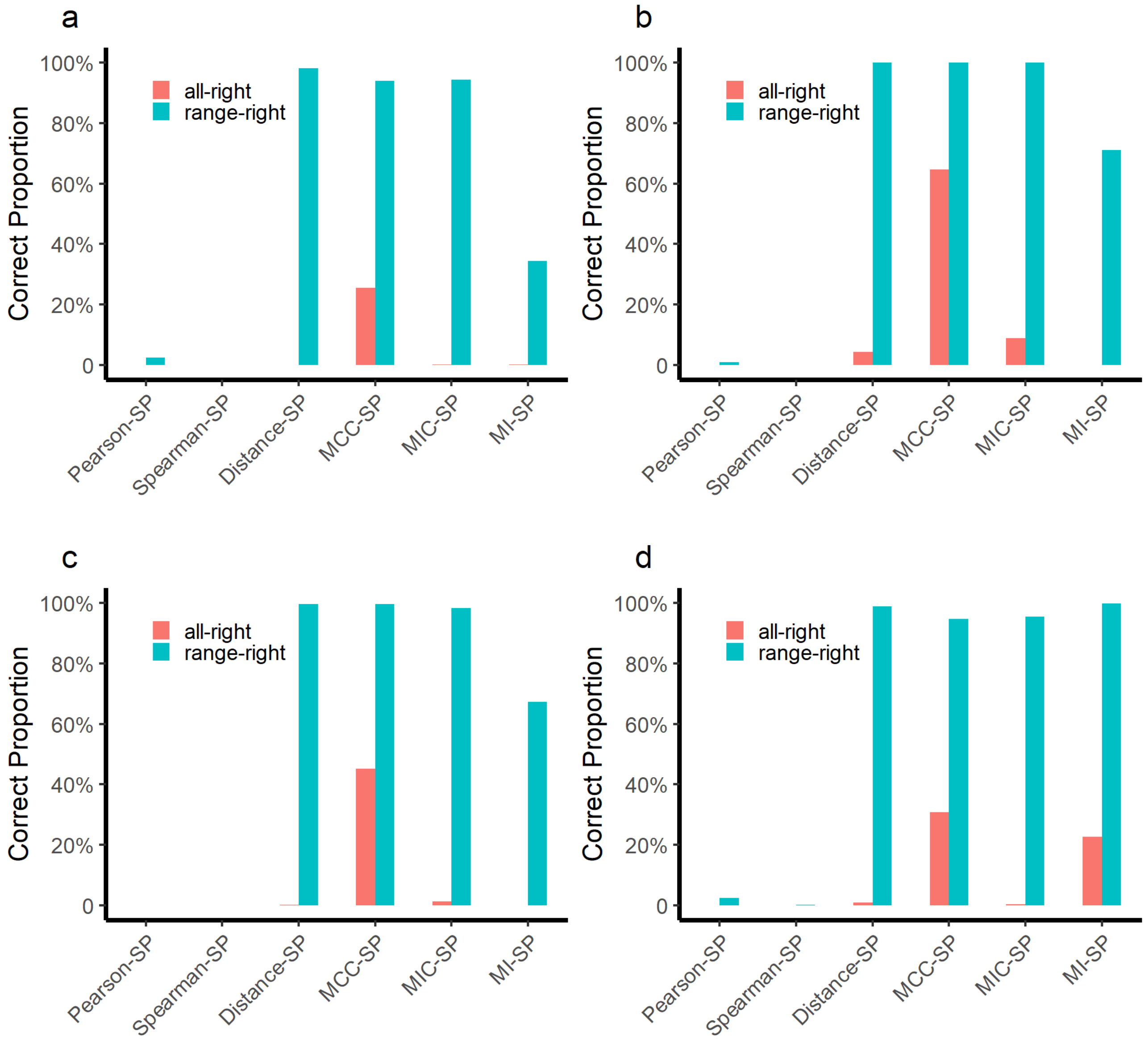

Figure S2. The proportion correctly pinpointing top 4 pathways under sample size 500 and nonlinear proportion 30%. The nonlinear pattern is (a) $\boldsymbol{\varphi(x_i) = \sqrt{C - x_i^2} + \varepsilon}$, (b) $\boldsymbol{\varphi(x_i) = \cos(x_i) + \varepsilon}$, (c) mixed nonlinear pattern (5 edges having cosine and 2 edges having quadratic relationship) and (d) $\boldsymbol{\varphi(x_i) = x_i^2 + \varepsilon}$ respectively.

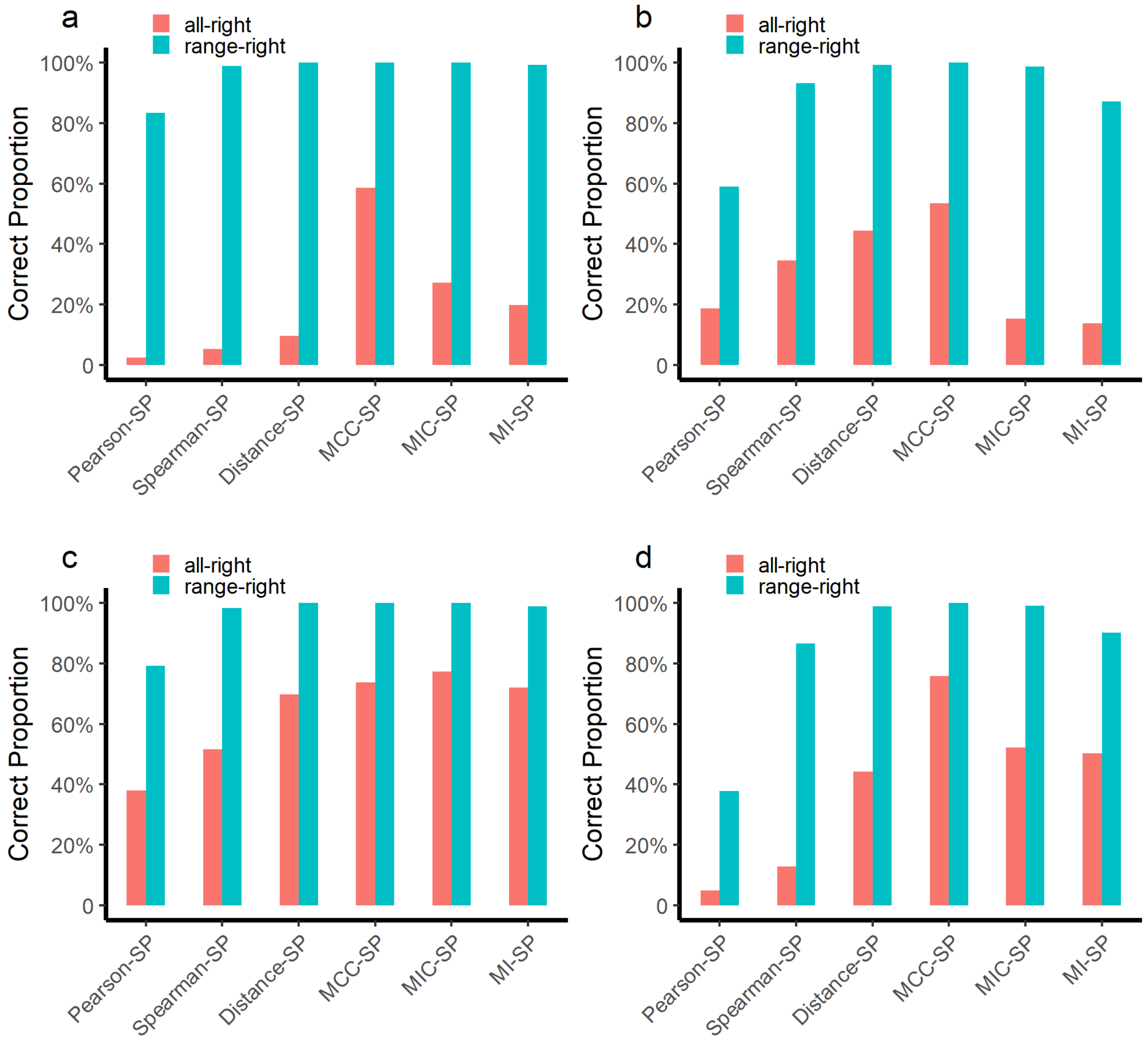

Figure S3. The proportion correctly pinpointing top 4 pathways under sample size 500 and nonlinear pattern being $\boldsymbol{\varphi(x_i) = \sin(2x_i) + \varepsilon}$. The proportion of nonlinear components is **(a)** 30%, **(b)** 40%, **(c)** 50% and **(d)** 60% respectively.

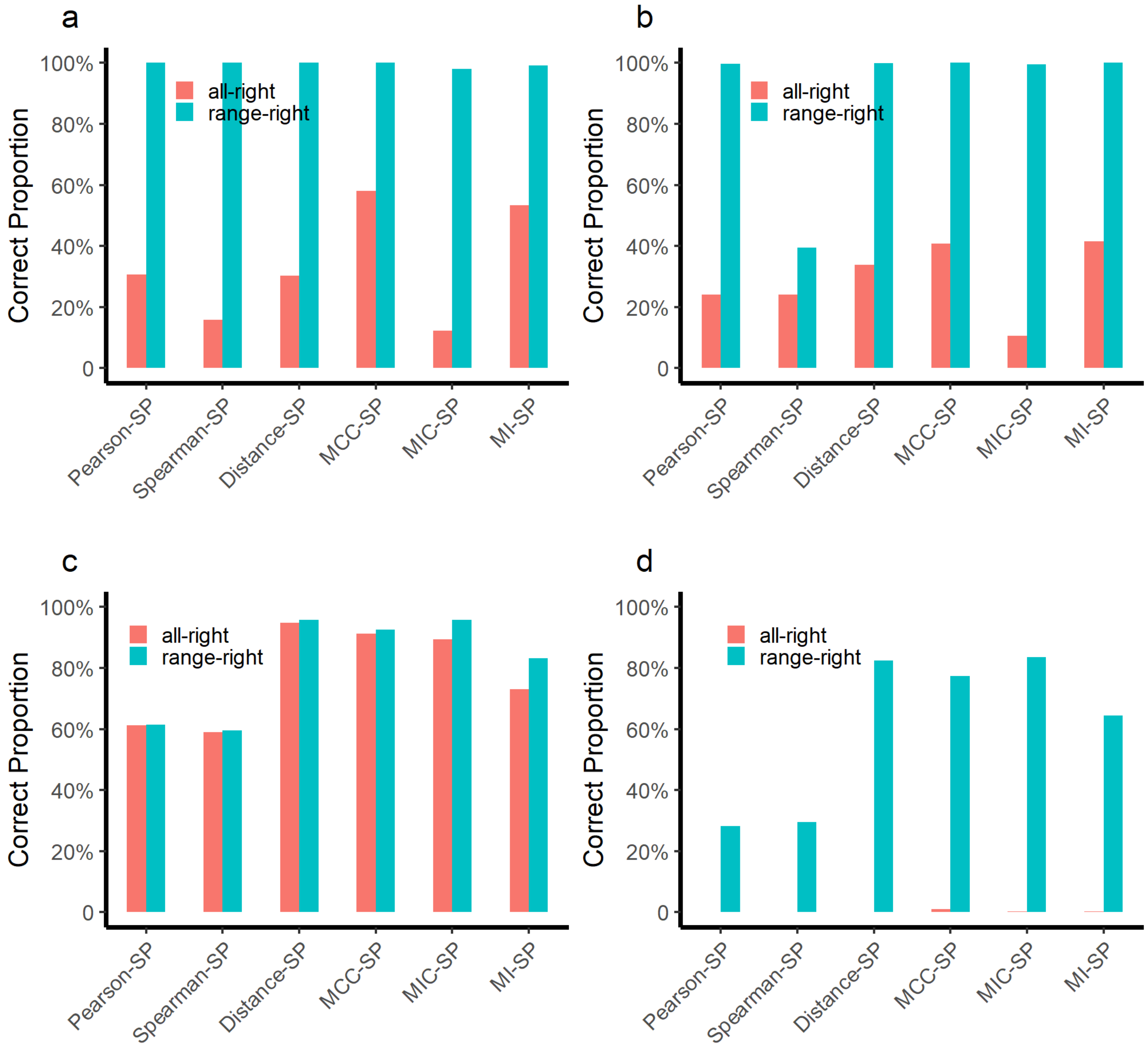

Figure S4. The proportion correctly pinpointing top 4 pathways under sample size 500. The nonlinear pattern is **(a)** $\boldsymbol{\varphi(x_i) = exp(x_i) + \varepsilon}$ under nonlinear proportion 40%, **(b)** $\boldsymbol{\varphi(x_i) = exp(x_i) + \varepsilon}$ under nonlinear proportion 50%, **(c)** $\boldsymbol{\varphi(x_i) = \frac{1}{x_i} + \varepsilon}$ under nonlinear proportion 40%, **(d)** $\boldsymbol{\varphi(x_i) = \frac{1}{x_i} + \varepsilon}$ under nonlinear proportion 50%.

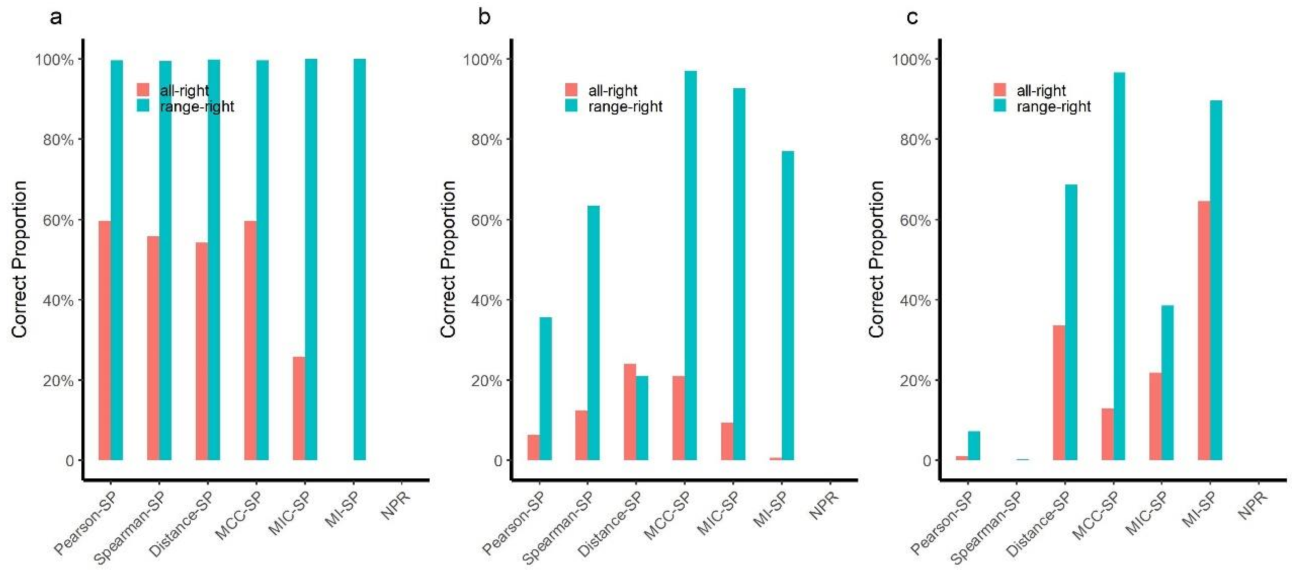

Figure S5. The proportion that correctly pinpoint the top 4 pathways among 500 simulations under two criteria when the sample size is 500 and the proportion of nonlinear components is 40%. **(a) linear correlation pattern**, **(b)** $\boldsymbol{\varphi(x_i) = \sin(2x_i) + \varepsilon}$, and **(c)** $\boldsymbol{\varphi(x_i) = x_i^2 + \varepsilon}$ respectively.

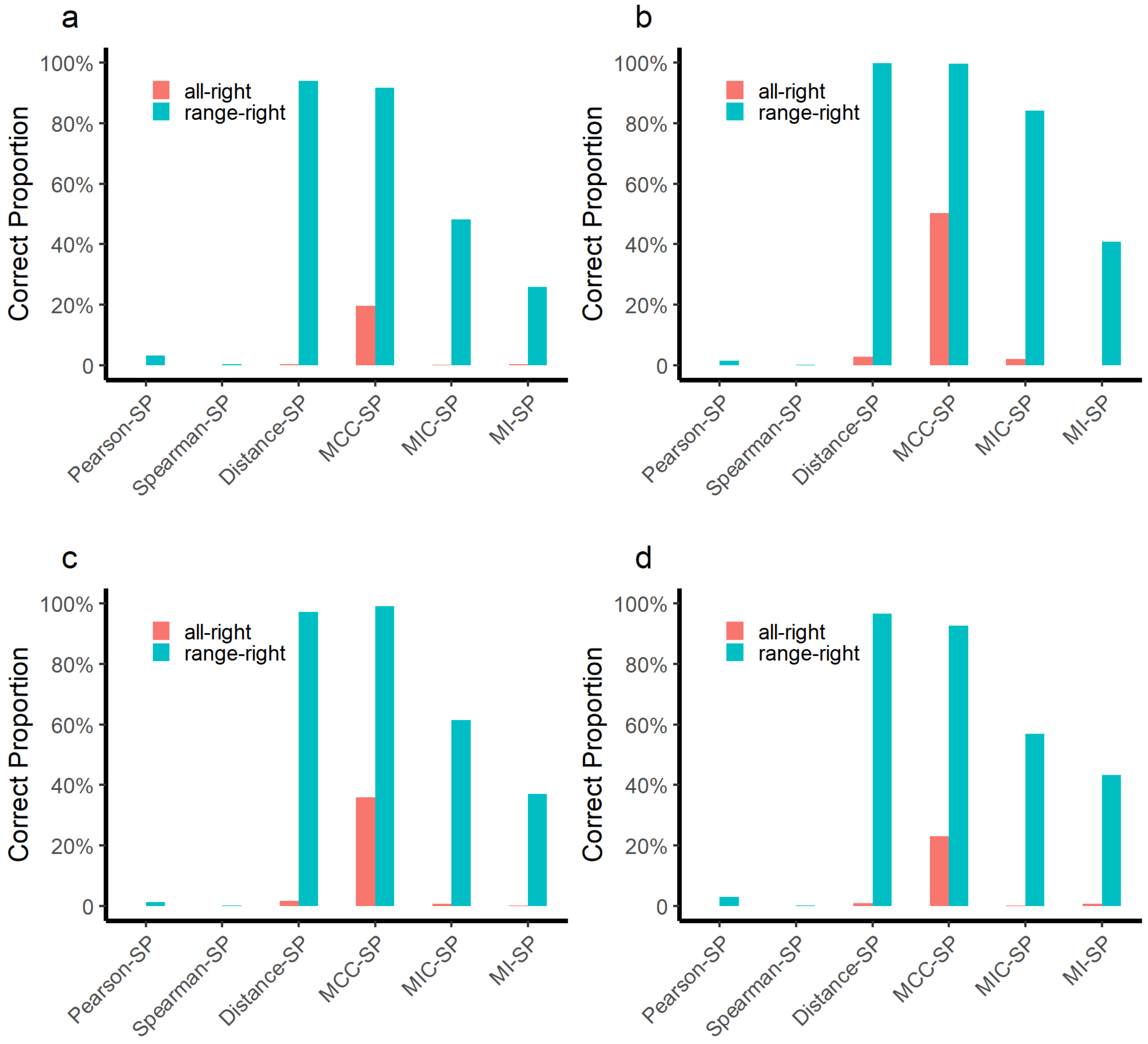

Figure S6. The proportion correctly pinpointing top 4 pathways under sample size 300 and nonlinear proportion 30%. The nonlinear pattern is **(a)** $\boldsymbol{\varphi(x_i) = \sqrt{C - x_i^2} + \varepsilon}$, **(b)** $\boldsymbol{\varphi(x_i) = \cos(x_i) + \varepsilon}$, **(c)** mixed nonlinear pattern (5 edges having cosine and 2 edges having quadratic relationship) and **(d)** $\boldsymbol{\varphi(x_i) = x_i^2 + \varepsilon}$ respectively.

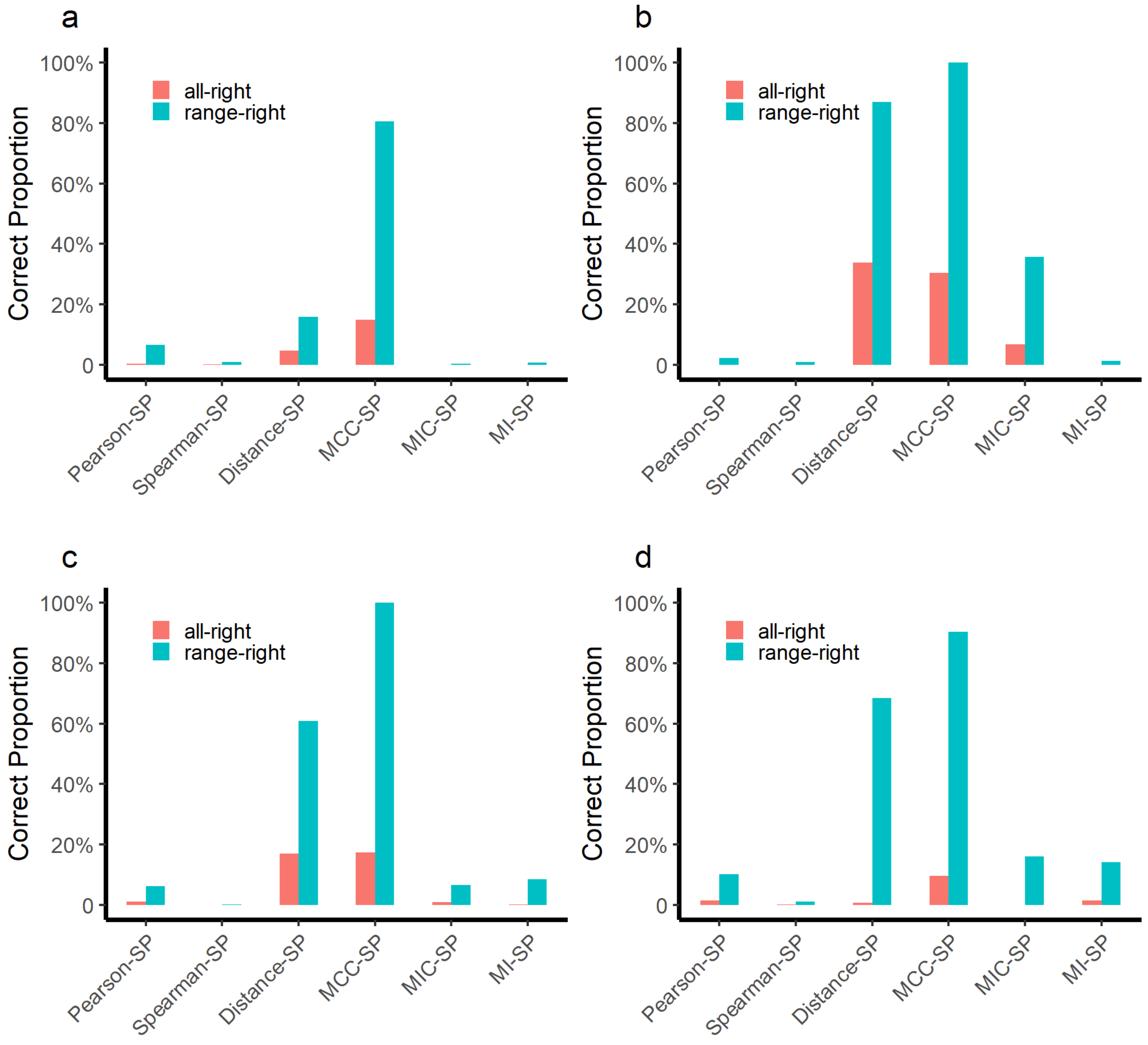

Figure S7. The proportion correctly pinpointing top 4 pathways under sample size 300 and nonlinear proportion 40%. The nonlinear pattern is **(a)** $\boldsymbol{\varphi(x_i) = \sqrt{C - x_i^2} + \varepsilon}$, **(b)** $\boldsymbol{\varphi(x_i) = \cos(x_i) + \varepsilon}$, **(c)** mixed nonlinear pattern (6 edges having cosine and 3 edges having quadratic relationship) and **(d)** $\boldsymbol{\varphi(x_i) = x_i^2 + \varepsilon}$ respectively.

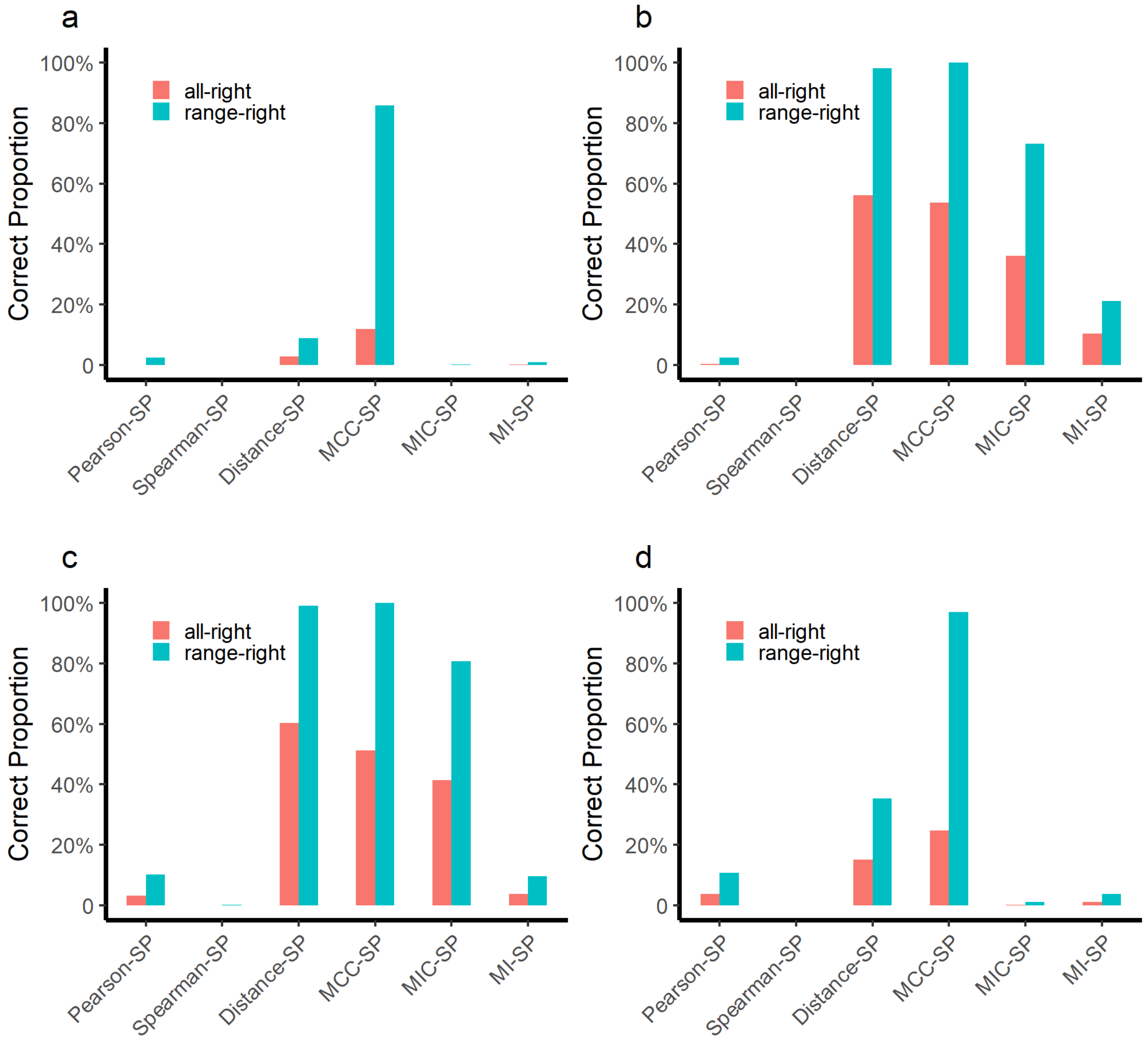

Figure S8. The proportion correctly pinpointing top 4 pathways under sample size 300 and nonlinear proportion 50%. The nonlinear pattern is **(a)** $\varphi(x_i) = \sqrt{C - x_i^2} + \varepsilon$, **(b)** $\varphi(x_i) = \cos(x_i) + \varepsilon$, **(c)** mixed nonlinear pattern (8 edges having cosine and 4 edges having quadratic relationship) and **(d)** $\varphi(x_i) = x_i^2 + \varepsilon$ respectively.

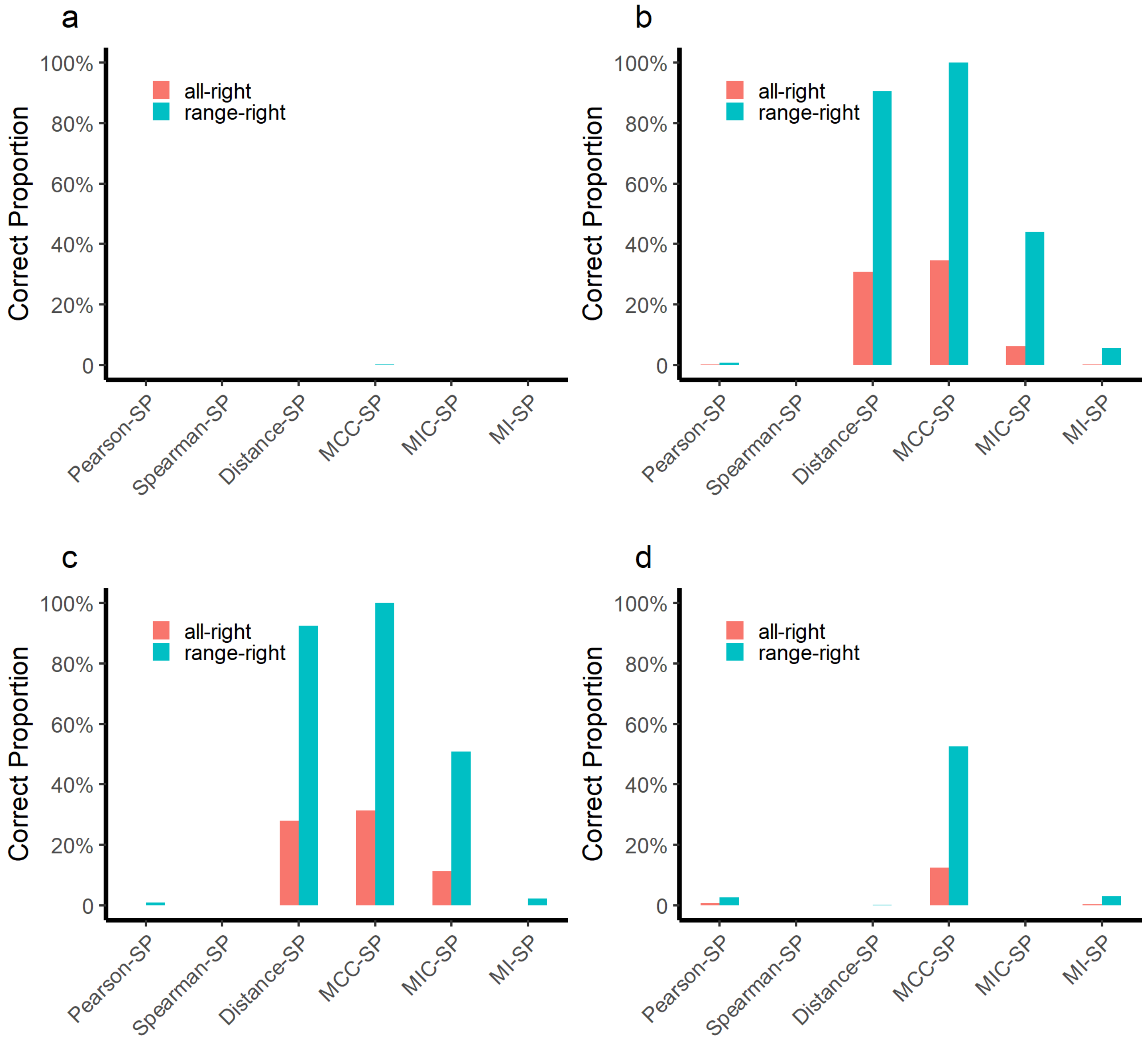

Figure S9. The proportion correctly pinpointing top 4 pathways under sample size 300 and nonlinear proportion 60%.The nonlinear pattern is **(a)** $\boldsymbol{\varphi(x_i) = \sqrt{C - x_i^2} + \varepsilon}$, **(b)** $\boldsymbol{\varphi(x_i) = \cos(x_i) + \varepsilon}$, **(c)** mixed nonlinear pattern (8 edges having cosine and 5 edges having quadratic and 1 edge having arcuate relationship) and **(d)** $\boldsymbol{\varphi(x_i) = x_i^2 + \varepsilon}$ respectively.

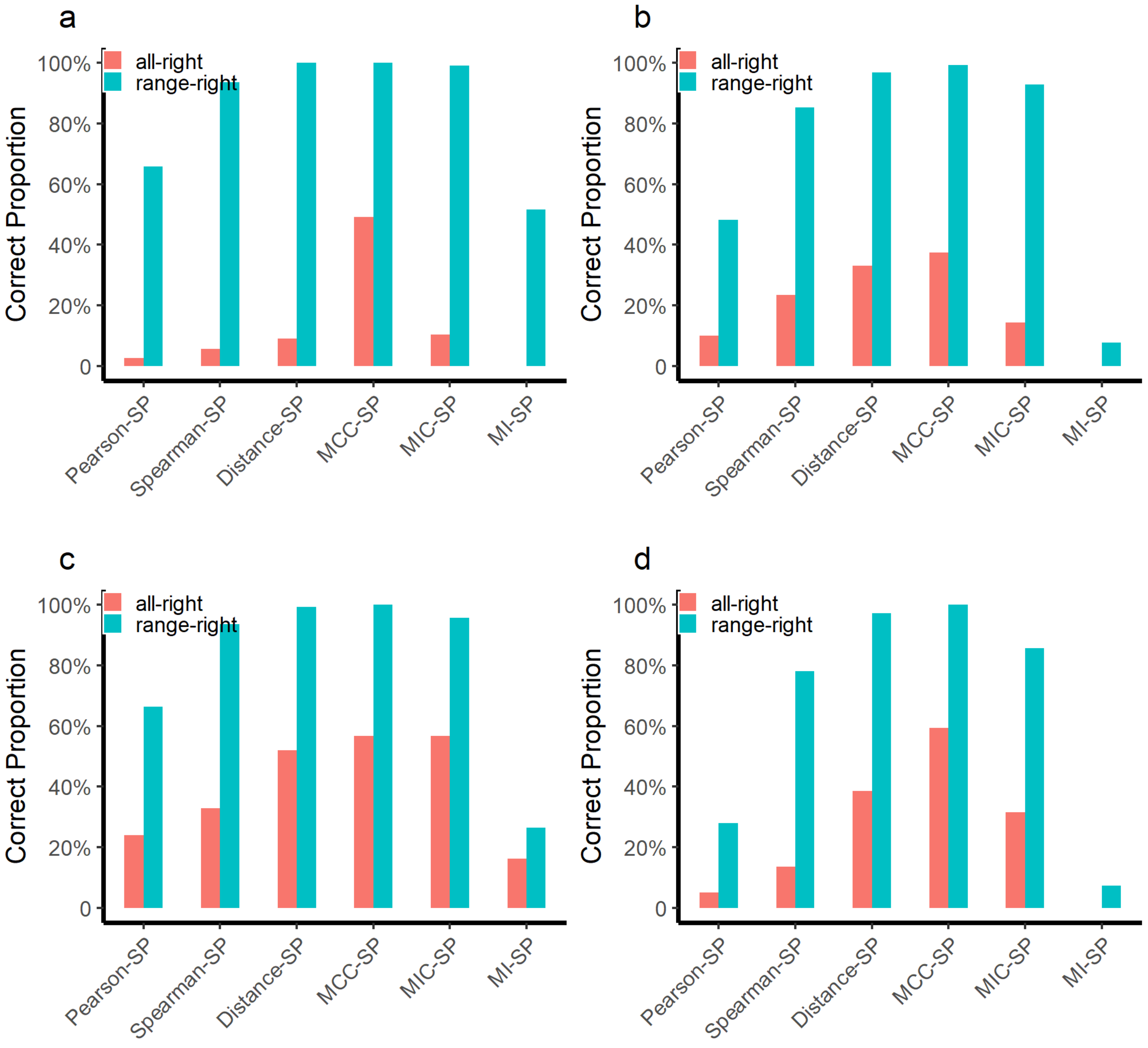

Figure S10. The proportion correctly pinpointing top 4 pathways under sample size 300 and nonlinear pattern being $\boldsymbol{\varphi}(\boldsymbol{x_i}) = \mathbf{sin}(\mathbf{2}\boldsymbol{x_i}) + \boldsymbol{\varepsilon}$. The proportion of nonlinear components is **(a)** 30%, **(b)** 40%, **(c)** 50% and **(d)** 60% respectively.

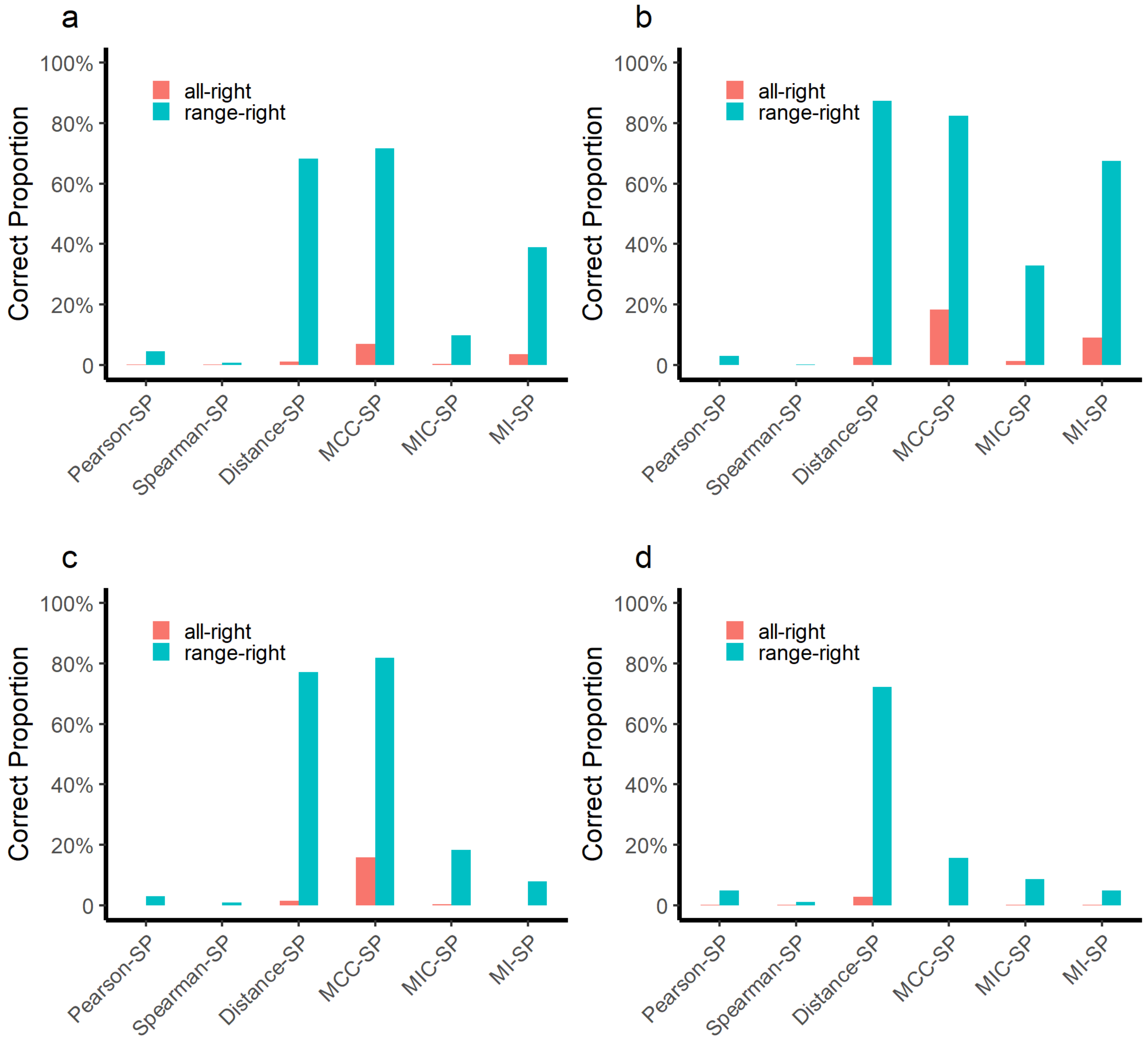

Figure S11. The proportion correctly pinpointing top 4 pathways under sample size 100 and nonlinear proportion 30%. The nonlinear pattern is **(a)** $\boldsymbol{\varphi(x_i) = \sqrt{C - x_i^2} + \varepsilon}$, **(b)** $\boldsymbol{\varphi(x_i) = \cos(x_i) + \varepsilon}$, **(c)** mixed nonlinear pattern (5 edges having cosine and 2 edges having quadratic relationship) and **(d)** $\boldsymbol{\varphi(x_i) = x_i^2 + \varepsilon}$ respectively.

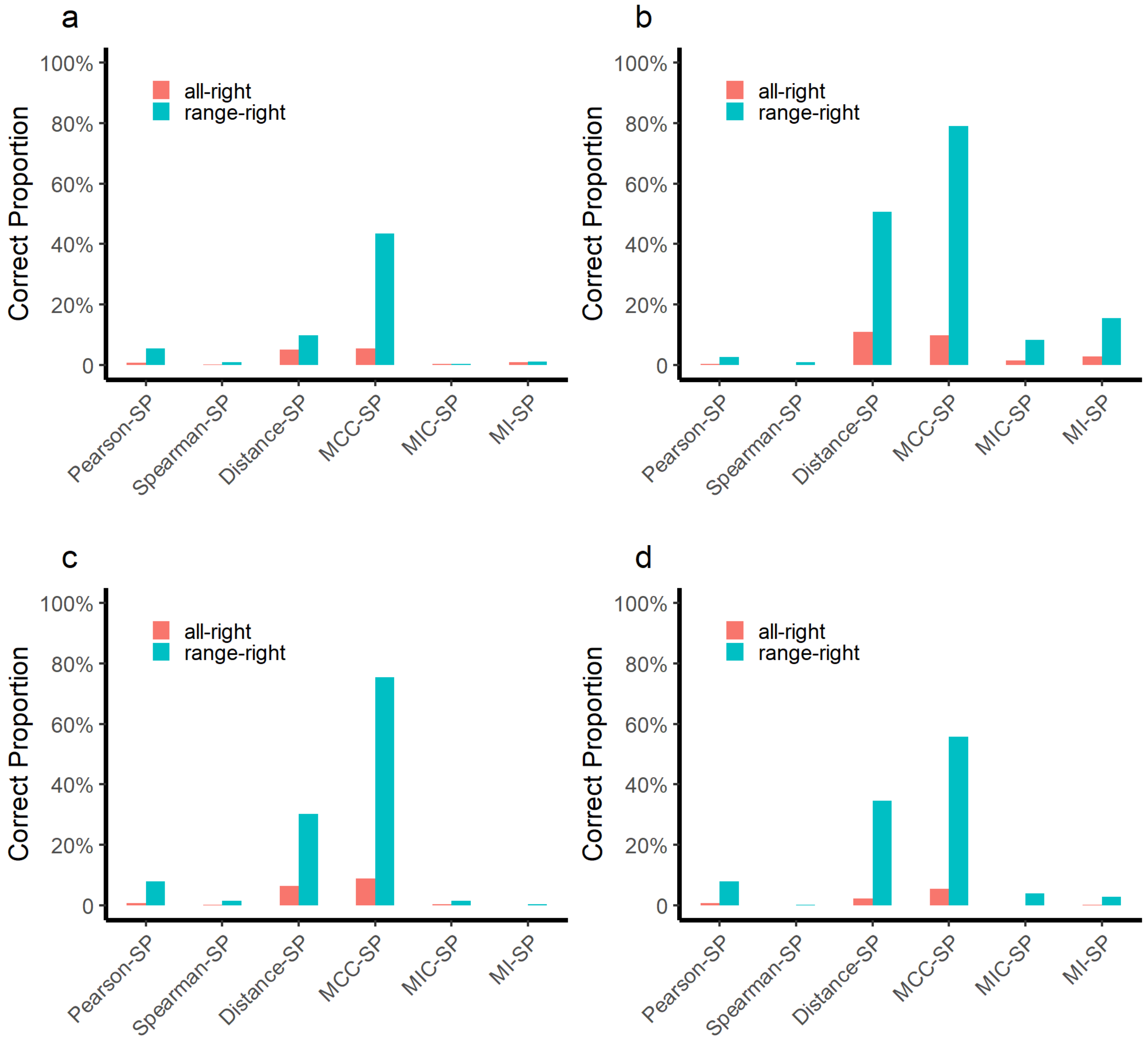

Figure S12. The proportion correctly pinpointing top 4 pathways under sample size 100 and nonlinear proportion 40%. The nonlinear pattern is **(a)** $\boldsymbol{\varphi(x_i) = \sqrt{C - x_i^2} + \varepsilon}$, **(b)** $\boldsymbol{\varphi(x_i) = \cos(x_i) + \varepsilon}$, **(c)** mixed nonlinear pattern (6 edges having cosine and 3 edges having quadratic relationship) and **(d)** $\boldsymbol{\varphi(x_i) = x_i^2 + \varepsilon}$ respectively.

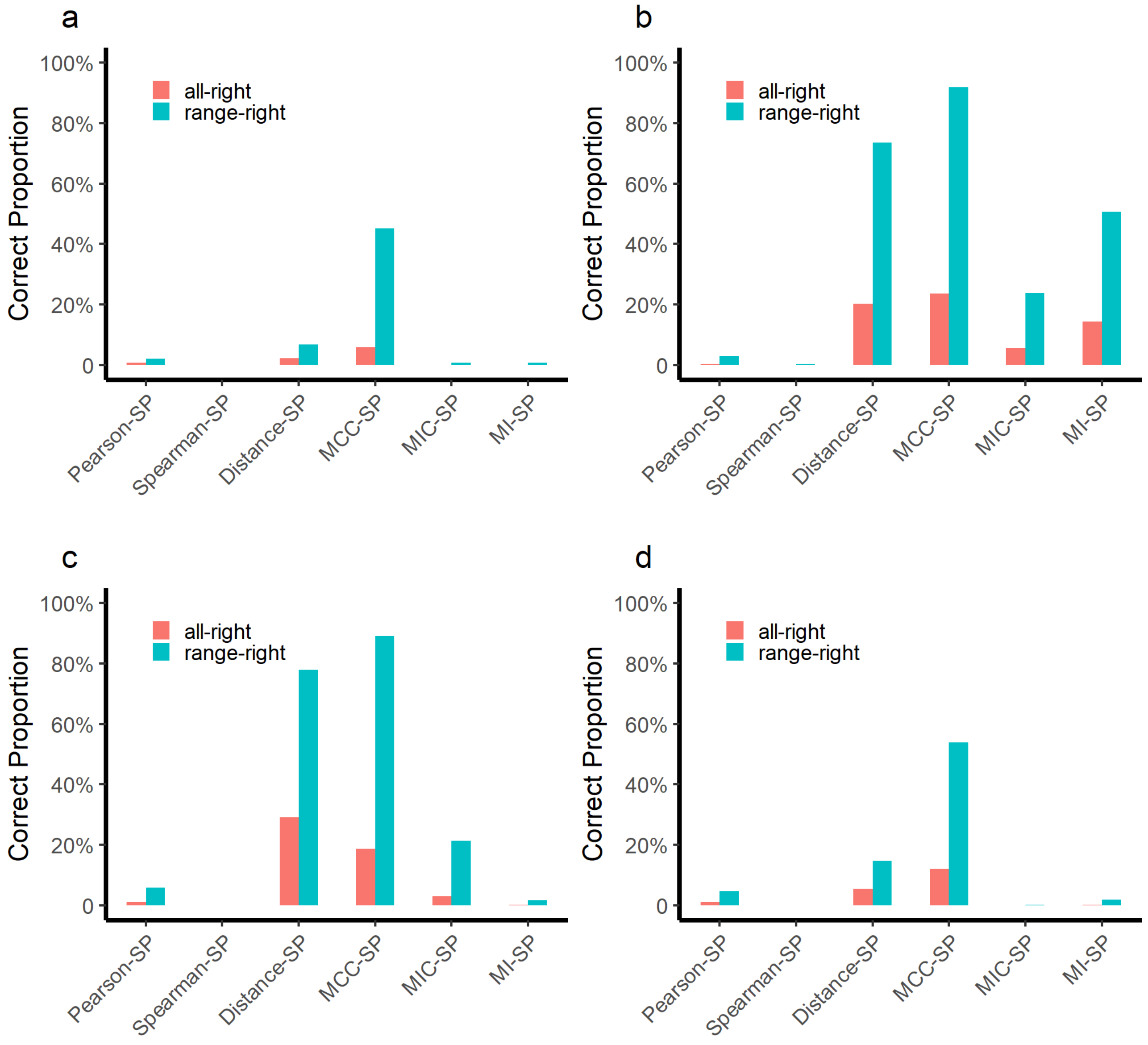

Figure S13. The proportion correctly pinpointing top 4 pathways under sample size 100 and nonlinear proportion 50%. he nonlinear pattern is **(a)** $\boldsymbol{\varphi(x_i) = \sqrt{C - x_i^2} + \varepsilon}$, **(b)** $\boldsymbol{\varphi(x_i) = \cos(x_i) + \varepsilon}$, **(c)** mixed nonlinear pattern (8 edges having cosine and 4 edges having quadratic relationship) and **(d)** $\boldsymbol{\varphi(x_i) = x_i^2 + \varepsilon}$ respectively.

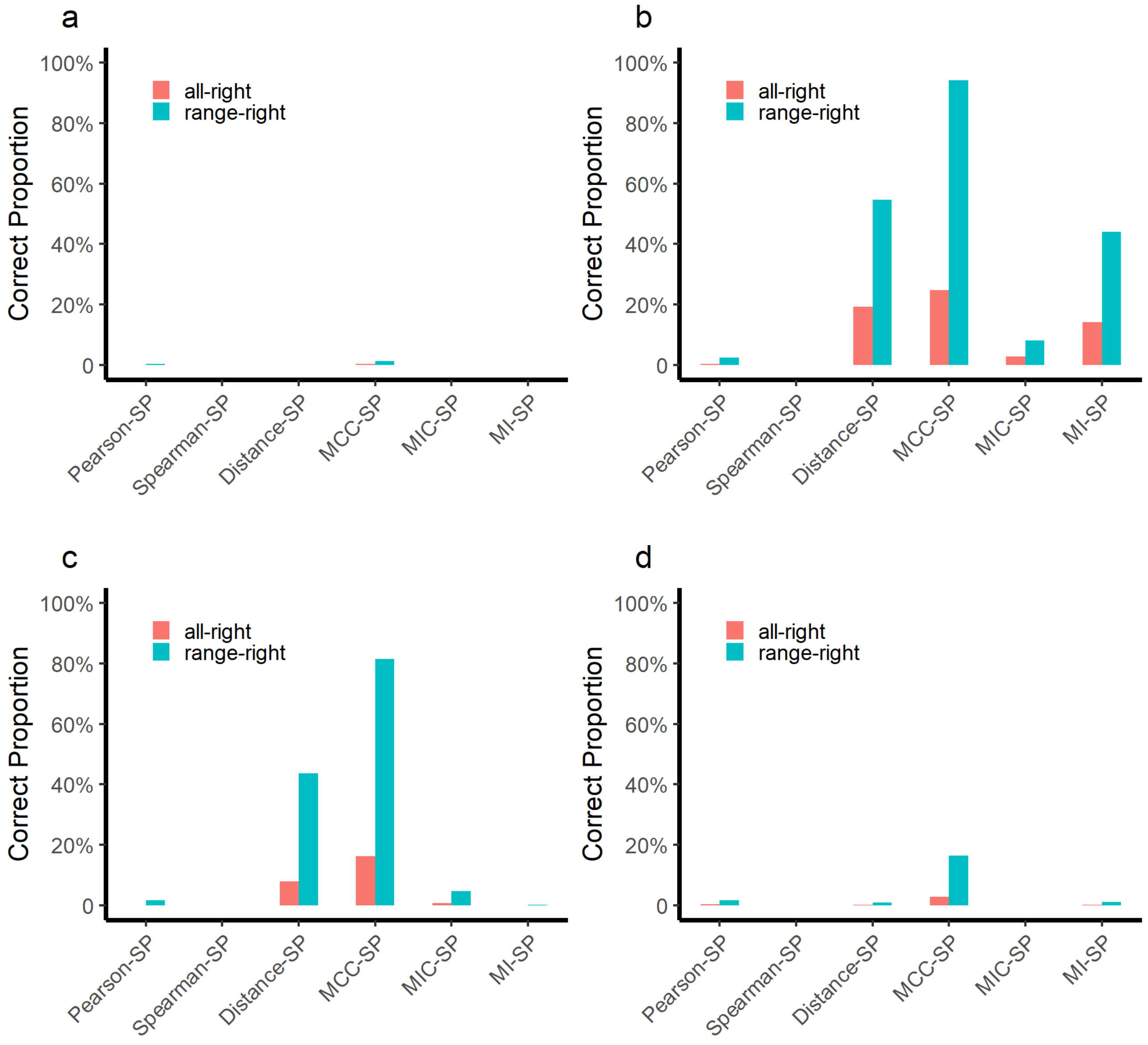

Figure S14. The proportion correctly pinpointing top 4 pathways under sample size 100 and nonlinear proportion 60%. The nonlinear pattern is **(a)** $\boldsymbol{\varphi(x_i) = \sqrt{C - x_i^2} + \varepsilon}$, **(b)** $\boldsymbol{\varphi(x_i) = \cos(x_i) + \varepsilon}$, **(c)** mixed nonlinear pattern (8 edges having cosine and 5 edges having quadratic and 1 edge having arcuate relationship) and **(d)** $\boldsymbol{\varphi(x_i) = x_i^2 + \varepsilon}$ respectively.

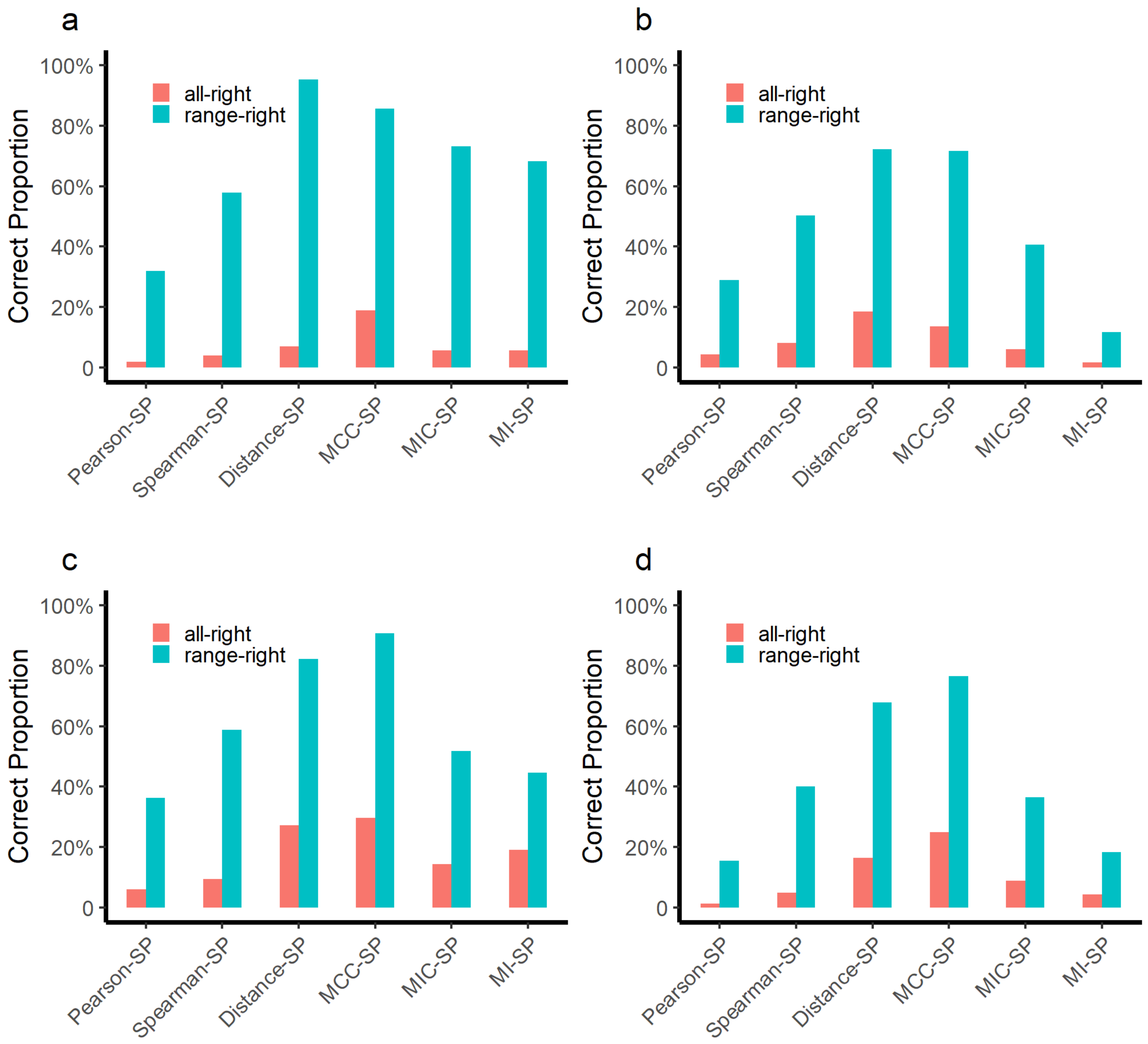

Figure S15. The proportion correctly pinpointing top 4 pathways under sample size 100 and nonlinear pattern being $\boldsymbol{\varphi(x_i) = \sin(2x_i) + \varepsilon}$ .The proportion of nonlinear components is **(a)** 30%, **(b)** 40%, **(c)** 50% and **(d)** 60% respectively.

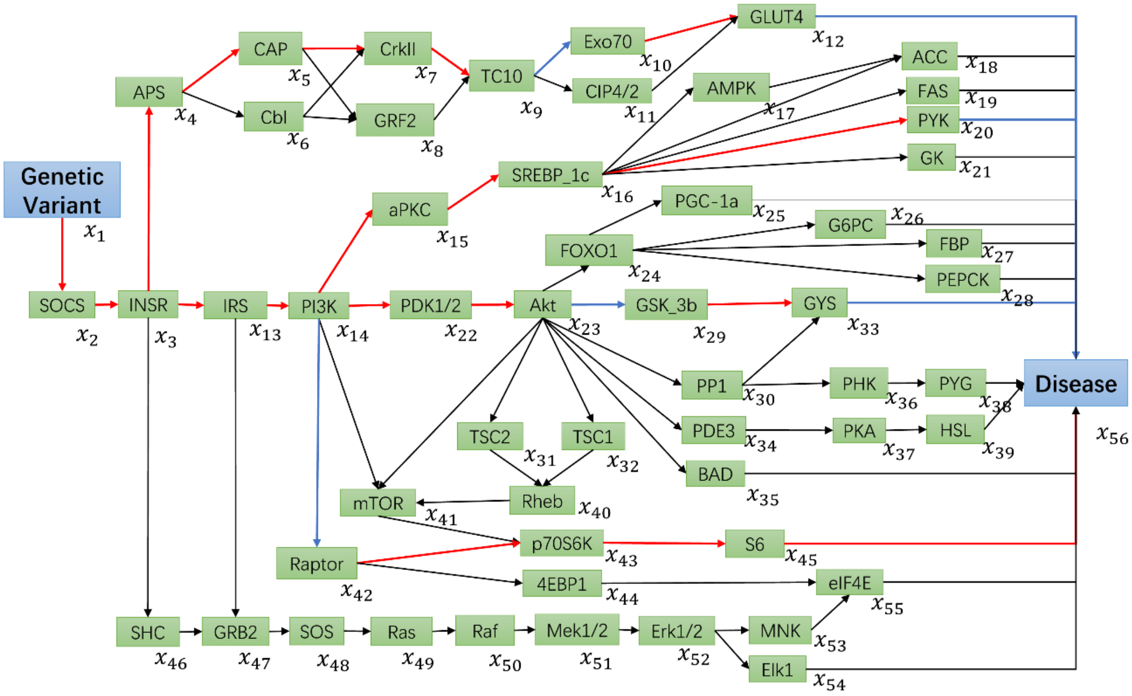

Figure S16. The network when there are 30% nonlinear between-node connection in the 4 effective pathways. The 4 effective pathways are highlighted in red with the non-linear correlation edge covered in blue.

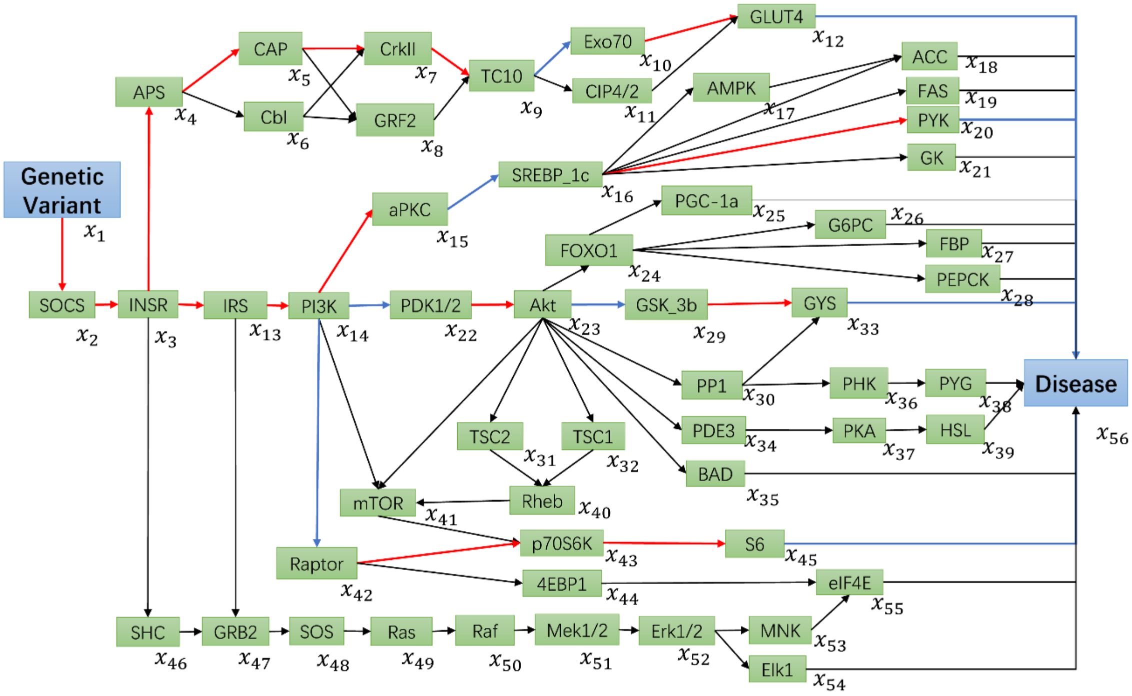

Figure S17. The network when there are 40% nonlinear between-node connection in the 4 effective pathways. The 4 effective pathways are highlighted in red with the non-linear correlation edge covered in blue.

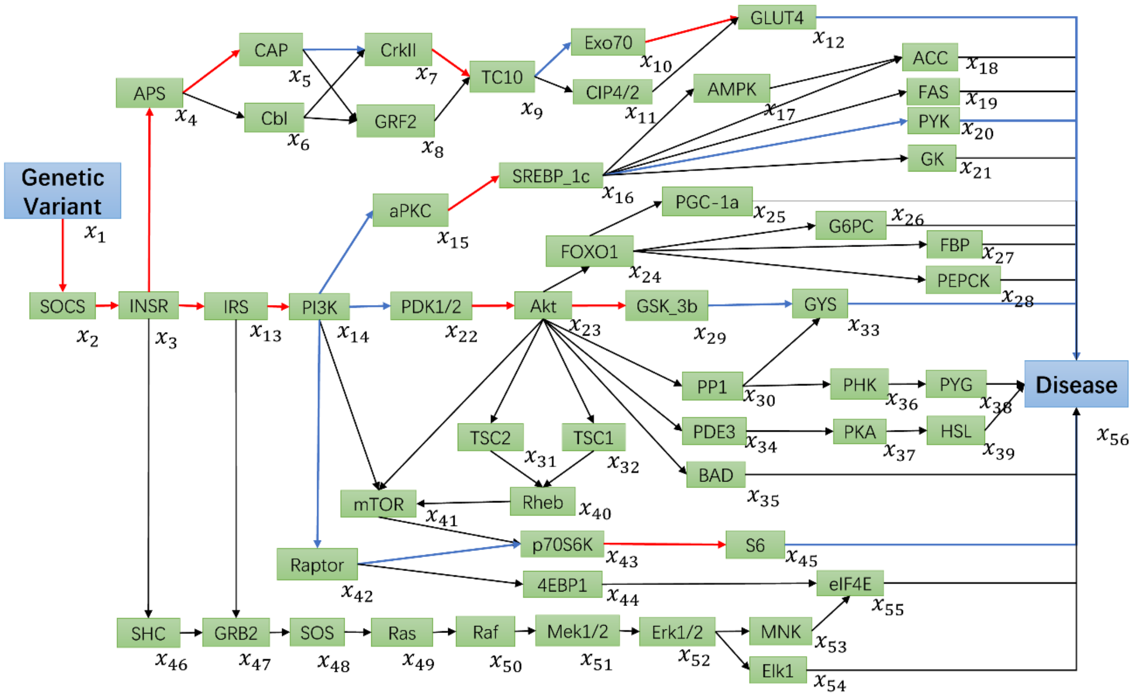

Figure S18. The network when there are 50% nonlinear between-node connection in the 4 effective pathways. The 4 effective pathways are highlighted in red with the non-linear correlation edge covered in blue.

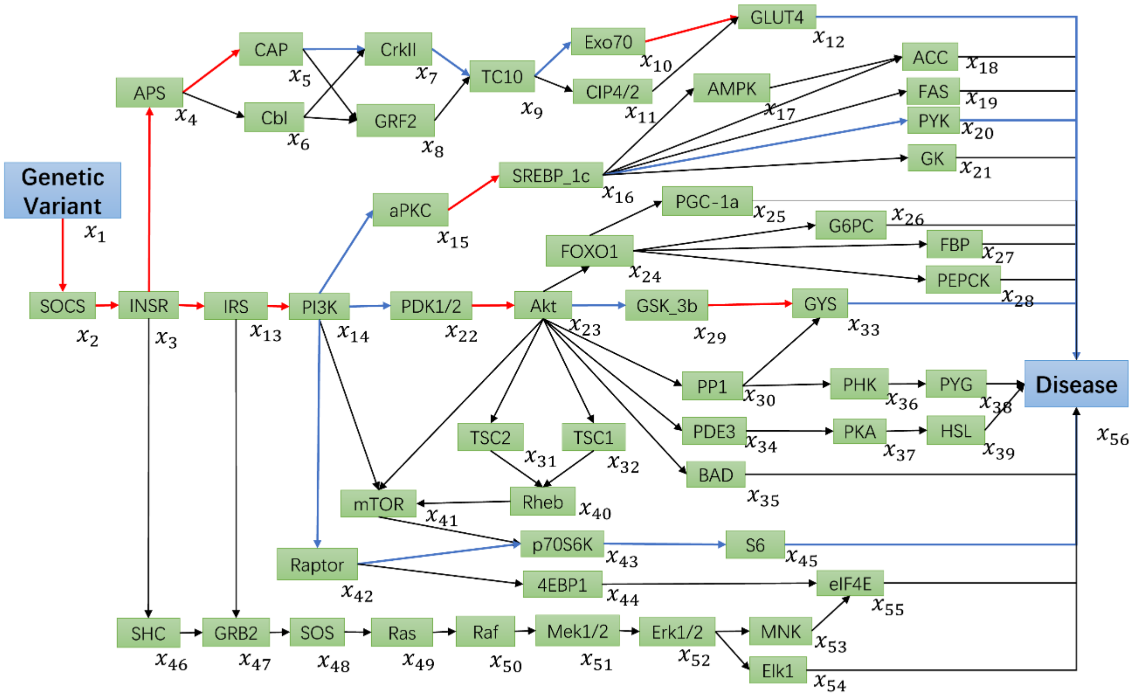

Figure S19. The network when there are 60% nonlinear between-node connection in the 4 effective pathways. The 4 effective pathways are highlighted in red with the non-linear correlation edge covered in blue.

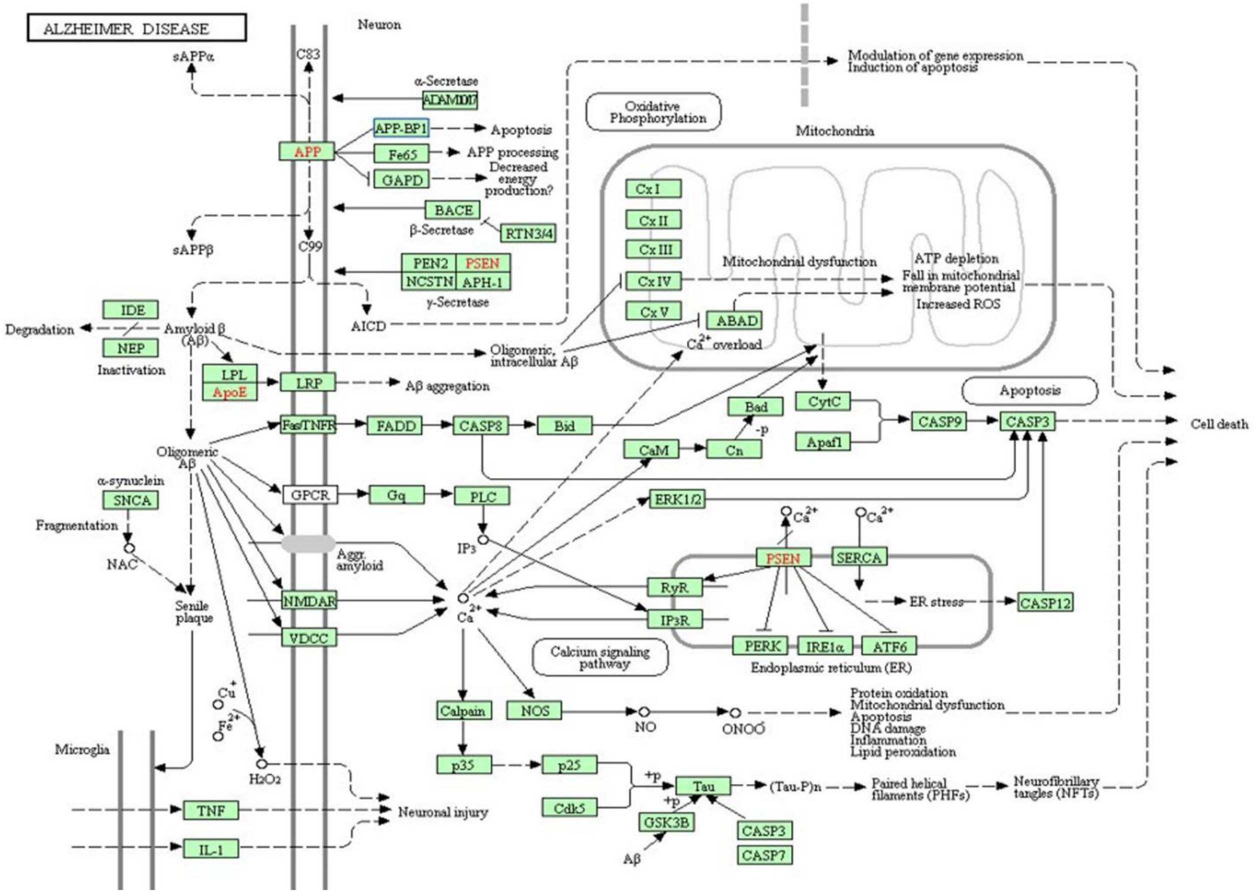

Figure S20. The Alzheimer disease pathway downloaded from Kyoto Encyclopedia of Genes and Genomes (KEGG)
